# Proteolytic processing of the L-type Ca ^2+^ channel alpha _1_1.2 subunit in neurons

**DOI:** 10.12688/f1000research.11808.2

**Published:** 2018-08-17

**Authors:** Olivia R. Buonarati, Peter B. Henderson, Geoffrey G. Murphy, Mary C. Horne, Johannes W. Hell

**Affiliations:** 1 Department of Pharmacology, University of California, Davis, CA, USA; 2 Department of Molecular and Integrative Physiology, Molecular and Behavioral Neuroscience Institute, University of Michigan, Ann Arbor, MI, USA

**Keywords:** Cav1.2, calpain cleavage, neuronal calcium

## Abstract

**Background**: The L-type Ca2+ channel Cav1.2 is a prominent regulator of neuronal excitability, synaptic plasticity, and gene expression. The central element of Cav1.2 is the pore-forming α _1_1.2 subunit. It exists in two major size forms, whose molecular masses have proven difficult to precisely determine. Recent work suggests that α _1_1.2 is proteolytically cleaved between the second and third of its four pore-forming domains (Michailidis *et al*,. 2014).

**Methods**: To better determine the apparent molecular masses (M _R_)of the α _1_1.2 size forms, extensive systematic immunoblotting of brain tissue as well as full length and C-terminally truncated α _1_1.2 expressed in HEK293 cells was conducted using six different region–specific antibodies against α _1_1.2.

**Results**: The full length form of α _1_1.2 migrated, as expected, with an apparent M _R_ of ~250 kDa. A shorter form of comparable prevalence with an apparent M _R_ of ~210 kDa could only be detected in immunoblots probed with antibodies recognizing α _1_1.2 at an epitope 400 or more residues upstream of the C-terminus.

**Conclusions**: The main two size forms of α _1_1.2 are the full length form and a shorter form, which lacks ~350 distal C-terminal residues. Midchannel cleavage as suggested by Michailidis *et al*. (2014) is at best minimal in brain tissue.

## Introduction

L-type Ca ^2+^ channels are critical regulators of neuronal excitability ( Berkefeld *et al.*, 2006; Marrion & Tavalin, 1998), gene expression ( Dolmetsch *et al.*, 2001; Graef *et al.*, 1999; Li *et al.*, 2012; Ma *et al.*, 2014; Marshall *et al.*, 2011; Murphy *et al.*, 2014; Wheeler *et al.*, 2012), long-term potentiation (LTP) ( Boric *et al.*, 2008; Grover & Teyler, 1990; Moosmang *et al.*, 2005; Patriarchi *et al.*, 2016; Qian *et al.*, 2017), long-term depression (LTD) ( Bernard *et al.*, 2014; Bolshakov & Siegelbaum, 1994), and memory consolidation ( White *et al.*, 2008). Ca _v_1.2 is the most abundant L-type channel in the brain and heart ( Hell *et al.*, 1993a; Sinnegger-Brauns *et al.*, 2004). The multitude of Ca _v_1.2-dependent functions is illustrated by diseases such as Timothy Syndrome, which arises from one of three single missense mutations in exon 8/8A of the *CACNA1C* gene encoding the central, ion-conducting α _1_1.2 subunit. Symptoms of this rare autosomal dominant disorder manifest as syndactyly, autistic-like behaviors, and widespread organ dysfunctions including dysregulation of cardiac contractility and heart rate ( Splawski *et al.*, 2004).

The central subunit of Ca _v_1.2 that forms the ion-conducting pore, α _1_1.2, exists in two major size forms with molecular masses estimated to be between 230-250 and 190-210 kDa ( Bunemann *et al.*, 1999; Davare *et al.*, 1999; Davare & Hell, 2003; Davare *et al.*, 2000; De Jongh *et al.*, 1996; Hell *et al.*, 1996; Hell *et al.*, 1993a; Hell *et al.*, 1995; Hell *et al.*, 1993b; Kochlamazashvili *et al.*, 2010; Patriarchi *et al.*, 2016; Qian *et al.*, 2017). *CACNA1C* was first cloned from rabbit heart, where full length α _1_1.2 consists of 2171 residues with a predicted M _R_ of 243 kDa ( Mikami *et al.*, 1989). Differential splicing of exons encoding the N-terminus of α _1_1.2 and a number of other *CACNA1C* exons can result in isoforms that vary by 30 or more residues in length ( Liao *et al.*, 2015; Snutch *et al.*, 1991, and references therein). Determination of the precise sizes of these α _1_1.2 variants by SDS-PAGE is hampered by the fact that even a small increase in the concentration of acrylamide from 5 to 6 percent causes a strong change in migration of the two size forms ( Hell *et al.*, 1993b). These observations indicate that the migration behavior of α _1_1.2 during SDS-PAGE can be anomalous.

Several studies over the past two decades detail the regulatory importance of calpain-mediated proteolysis at the α _1_1.2 distal C-terminus (DCT) ( Fuller *et al.*, 2010; Hell *et al.*, 1996; Hell *et al.*, 1993b; Hulme *et al.*, 2006b). For instance, deletion of 300-470 residues from the C terminus resulted in a 4-6 fold increase in current density without an increase in gating currents when expressed in *Xenopus* oocytes ( Wei *et al.*, 1994). These findings suggest that the potentiation due to C-terminal deletions is not caused by increased surface expression of Ca _v_1.2, but by an increase in coupling of depolarization-induced movement of the voltage sensors to pore opening ( Wei *et al.*, 1994). Similarly, truncating α _1_1.2 after residues 1733, 1821, 1905, and 2024 increased current density in HEK293-derived tsA201 cells by several fold, which was reversed by co-expression or injection of distal fragments as separate polypeptides ( Fuller *et al.*, 2010; Gao *et al.*, 2001; Hulme *et al.*, 2006b). Further deletions at or before residue 1623 abrogated channel currents, consistent with earlier work identifying residues 1623-1666 as critical for Ca _v_1.2 surface expression ( Gao *et al.*, 2000). These latter findings are also in agreement with recent observations, in which binding of α-actinin to this region is important for Ca _v_1.2 surface expression ( Hall *et al.*, 2013; Tseng *et al.*, 2017).

Earlier evidence indicates that the 190-210 kDa short form results from proteolytic processing of the long form by the Ca ^2+^-stimulated protease calpain ( Hell *et al.*, 1996). More recent work has suggested that extensive proteolytic processing occurs via calpain- and ubiquitin/proteasome-mediated mechanisms that target the intracellular loop between domains II and III, yielding two prominent α _1_1.2 fragments: a 90 kDa fragment that might consist of the N-terminus and the first two integral membrane domains I and II, and a 150 kDa fragment that might consist of domains III and IV and the long C-terminus ( Michailidis *et al.*, 2014).

We performed a long overdue, systematic analysis of α _1_1.2 size forms using region-specific antibodies, increasing concentrations of acrylamide, and surface biotinylation to examine their migration behavior during SDS-PAGE. As expected, one of the two main size forms of α _1_1.2 migrates according to an apparent M _R_ of 250 kDa, corresponding very well with the predicted size of the full length subunit. Importantly our study also provides very consistent and clear evidence that extensive proteolytic processing of α _1_1.2 occurs within the last ~660 C-terminal residues, with minimal cleavage in the middle of the pore-forming portion of the channel. Although removal of the DCT would be expected to increase channel currents ( Fuller *et al.*, 2010; Wei *et al.*, 1994), the severed DCT remains associated with the main channel portion to maintain a reduction of channel activity ( Fuller *et al.*, 2010; Gao *et al.*, 2001; Hulme *et al.*, 2006b).

## Materials and methods

### Animals

We used 6–12 week old 50% C57black/6N and 50% 129Sv hybrid mice (Jackson Laboratories, Bar Harbor, MN), α _1_1.2 conditional knockout (cKO) mice and their litter-matched WT controls as described ( Patriarchi *et al.*, 2016; White *et al.*, 2008), and 8–12 week old Sprague Dawley rats (Harlan). Ca _V_1.2 cKO mice of neuron-specific deletion and their wild-type littermates were on a C57BL/6NTac:129SvEv F2 genetic background. Mice with a floxed Ca _V_1.2 exon two allele (Ca _V_1.2 ^f/+^ or Ca _V_1.2 ^f/f^) and maintained on a 129SvEv genetic background were first bred to transgenic mice expressing the Cre recombinase regulated by the synapsin 1 promoter (Syn1-Cre ^Cre/+^) and maintained on a C57BL/6NTac background ( Cui *et al.*, 2008; Zhu *et al.*, 2001), producing an F1 cross. Using non-littermate offspring from the F1 cross, heterozygous floxed, cre-positive (Ca _V_1.2 ^f/+^; Syn1-Cre ^Cre/+^) mice were then crossed with heterozygous floxed, cre-negative (Ca _V_1.2 ^f/+^; Syn1-Cre ^+/+^) mice to produce homozygous floxed, Cre-positive (Ca _V_1.2 ^f/f^; Syn1-Cre ^Cre/+^) conditional knockout mice as well as wild-type mice (Ca _V_1.2 ^+/+^; Syn1-Cre ^+/+^). All animals were housed by the Animal Care Unit in Tupper Hall at UC Davis. This facility is fully approved for NIH-funded research and accredited by the Association for Assessment and Accreditation of Laboratory Animal Care. It maintains animals in a highly controlled environment optimized for the comfort of rodents in accordance with the applicable portions of the Animal Welfare Act and the DHS “Guide to the Care and Use of Animals.” Its NIH Office of Laboratory Animal Welfare Assurance Number is A3433-01. All efforts were made to ameliorate any potential suffering of animals. Specifically, animals were anesthetized with 5% isoflurane for 2–3 minutes in a two-chamber drop jar before decapitation and collection of tissue. This procedure followed NIH guidelines and was approved by the Institutional Animal Care and Use Committees at the University of California at Davis.

### Antibodies

Residue numbers correspond to the initial α _1_1.2 sequence from rabbit heart (Gene Bank Accession number: CAA33546).

The polyclonal antibody CNC1 was produced against the synthetic peptide (KY)TTKINMDDLQPSENEDKS, covering residues 818 to 835 within the intracellular loop between domains II and III of α _1_1.2 ( Dubel *et al.*, 1992). The peptide was coupled to bovine serum albumin in the laboratory of W. A. Catterall (University of Washington, WA, USA) and used to immunize rabbits ( Hell *et al.*, 1993b). Before use, the antibody was affinity purified on the same peptide cross-linked to Sepharose 4B-CL (for validation and characterization of CNC1 see Davare *et al.*, 1999; Hall *et al.*, 2013; Hell *et al.*, 1993a; Hell *et al.*, 1993b). The lysine and tyrosine residues at the N-terminus had been added for cross-linking and labeling purposes.

The polyclonal antibody ACC-003 was obtained from the company Alomone Labs (catalog number ACC-003, batch number ACC003AN4725; Jerusalem, Israel). It was produced in rabbit against the synthetic peptide (C)TTKINMDDLQPSENEDKS, which like CNC1, covers residues 818 to 835 within the intracellular loop between domains II and III of α _1_1.2. The cysteine at the N-terminus is not part of the original α _1_1.2 sequence but had presumably been added for cross-linking purposes. The batch of this antibody we received was characterized in Figure 2.

The polyclonal antibody FP1 was produced against an N-terminal GST fusion protein covering residues 783 to 845 within the same intracellular loop between domains II and III of α _1_1.2 as CNC1. The affinity purified GST fusion protein was used to immunize rabbits in the laboratory of J. W. Hell (University of Wisconsin, WI, USA). Before use, the antibody was affinity purified on the same GST fusion protein cross-linked to glutathione Sepharose (for validation and characterization see Davare *et al.*, 2001; Davare *et al.*, 2000; Hall *et al.*, 2013; Hall *et al.*, 2007; Hall *et al.*, 2006).

The polyclonal antibody CNC2 antibody was produced against the synthetic peptide (KY)GRGQSEEALPDSRSYVS covering residues 2122-2138 of α _1_1.2, a region ~40 residues upstream of the very C terminus of α _1_1.2 ( Hell *et al.*, 1993b). The peptide was coupled to bovine serum albumin in the laboratory of W. A. Catterall (University of Washington, WA, USA) and used to immunize rabbits ( Hell *et al.*, 1993b). Before use, the antibody was affinity purified on the same peptide cross-linked to Sepharose 4B-CL (for validation and characterization see Davare *et al.*, 1999; Hall *et al.*, 2013; Hell *et al.*, 1996; Hell *et al.*, 1993b; Hulme *et al.*, 2006a). The lysine and tyrosine residues at the N-terminus had been added for cross-linking and labeling purposes.

The phosphospecific polyclonal antibody against pS1700 was produced against the synthetic peptide EIRRAIpSGDLTAEEEL (residues 1694-1713) ( Fuller *et al.*, 2010). The peptide was coupled to bovine serum albumin in the laboratory of W. A. Catterall (University of Washington, WA, USA) and used to immunize rabbits. Before use, the antibody was affinity purified on the same peptide cross-linked to Sepharose 4B-CL (for validation and characterization see Fuller *et al.*, 2010; Murphy *et al.*, 2014).

The phosphospecific polyclonal antibody against pS1928 was produced against the synthetic peptide LGRRApSFHLECLK (residues 1923-1932) ( Davare *et al.*, 1999). The peptide was coupled to bovine serum albumin in the laboratory of W. A. Catterall (University of Washington, WA, USA) and used to immunize rabbits. Before use, the antibody was affinity purified on the same peptide cross-linked to Sepharose 4B-CL (for validation and characterization see Davare & Hell, 2003; Davare *et al.*, 2000; Hall *et al.*, 2007; Hall *et al.*, 2006).

### Immunoprecipitation and Immunoblotting

All procedures were performed on ice. Instruments, including centrifuge rotors, tubes, tools, and buffers, were pre-cooled at 4°C or on ice to minimize post-mortem proteolysis ( Hell *et al.*, 1993a; Hell *et al.*, 1993b; Westenbroek *et al.*, 1992). Whole mouse brains and acute rat forebrain and cortical slices were extracted with 1% Triton X-100 in 150 mM NaCl, 10 mM EDTA, 10 mM EGTA, 10 mM Tris, pH 7.4 containing protease inhibitors (0.1 mM phenylmethylsulfonyl fluoride, 1 µM pepstatin A, 2 µM leupeptin, 4 µM aprotinin) and phosphatase inhibitors (2 µM microcystin LR, 1 mM p-nitrophenyl phosphate, 1 mM sodium pyrophosphate, 2.5 mM sodium fluoride). Extracts were cleared by 30 minutes centrifugation (250,000xg). The soluble fraction was incubated on a head-over-head tilter with protein A - Sepharose beads and 2 µg FP1 antibody for 4 h at 4° C and washed three times with 0.1% Triton X-100 in 150 mM NaCl, 10 mM EDTA, 10 mM EGTA, 10 mM Tris, pH 7.4. Immunoprecipitated Ca _v_1.2 underwent SDS-PAGE in gels with a stacking phase polymerized from 3.5% acrylamide and a separating phase polymerized from 5, 7, 9, 11, or 13% acrylamide. Protein was transferred to polyvinylidene fluoride (PVDF) membranes at 50 V for 600 minutes for subsequent probing as previously described ( Davare *et al.*, 1999; Hell *et al.*, 1993a; Hell *et al.*, 1993b). Briefly, membranes were blocked in 10% milk, incubated in affinity-purified primary antibody (FP1 1:800, CNC1 1:200, CNC2 1:50, anti-pS1700 1:400, anti-pS1928 1:100, ACC-003 1:400) for 2 hours, washed, incubated in horseradish peroxidase (HRP)-labeled Protein A for 1 hour, washed, and developed on autoradiography film using chemiluminescence.

### Biotinylation

Forebrain slices were prepared from rat brain, then non-cortical regions trimmed when indicated to obtain cortical slices, equilibrated in oxygenated (95% O _2_, 5% CO _2_) artificial cerebral spinal fluid (ACSF: 119 mM NaCl, 26 mM NaHCO _3_, 1.25 mM NaH _2_PO _4_, 2.5 mM KCl, 1 mM MgSO _4_, 2.2 mM CaCl _2_, 15 mM glucose, 1 mM myo-inositol, 2 mM Na-pyruvate, 0.4 mM ascorbic acid) at 32°C for 1 h, and labeled at 4°C for 45 min in 2 ml ACSF containing 1 mg/ml Sulfo-NHS-SS-biotin (Pierce). Oxygenation of all slices was maintained throughout the entirety of the experiment for slice equilibration, biotinylation, quenching and lysis procedures. Excess Sulfo-NHS-SS-biotin was quenched by washing slices four times with ice-cold ACSF buffer containing 100 mM glycine. Cells were homogenized on ice with 50 mM Tris-Cl pH 7.4, 150 mM NaCl, 10 mM EGTA, 10 mM EDTA, 1% NP-40, 10% Glycerol, 0.05% SDS, 0.4% DOC containing protease and phosphatase inhibitors and insoluble material removed by centrifugation (10,000 xg, 20 min). Biotinylated constituents in lysate, each containing 300 μg of protein, were affinity-purified by incubation with 30 µl of NeutrAvidin-conjugated Sepharose beads (Thermo-Fisher) for 3 h at 4°C. Following four ice-cold washes of bead-bound material with 1% Triton X-100, 150 mM NaCl, 10 mM Tris-Cl, 10 mM EDTA, 10 mM EGTA, immobilized proteins were eluted by treatment with SDS sample buffer, separated by SDS-PAGE (8% resolving gel), and transferred to PVDF before immunoblotting as above.

## Results

### Assignment of immunoreactive bands to α _1_1.2

**Figure 1.**
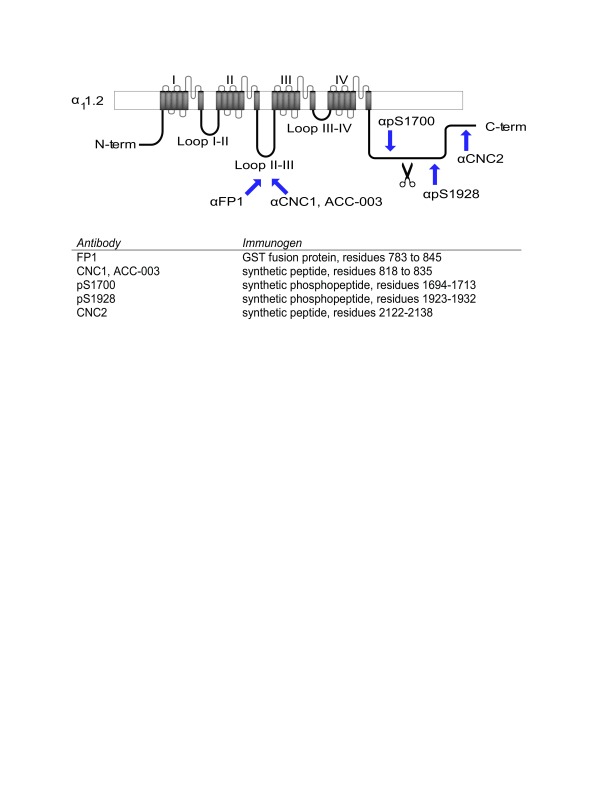
Location of antibody epitopes. Shown is a schematic of the Ca _v_1.2 α _1_1.2 subunit, in which regions used as immunogens for the depicted antibodies are identified by arrows. Exact residues are listed in the table and numbered according to α _1_1.2 given in Gene Bank Accession number CAA33546. FP1, CNC1, and ACC-003 are directed against the loop between domains II and III, pS1700 against phosphorylated S1700, pS1928 against phosphorylated S1928, and CNC2 against residues 2122-2138 of α _1_1.2, which are ~40 residues upstream of the very C terminus of α _1_1.2.

To identify the main size variants of brain α _1_1.2, we performed immunoblotting with three different antibodies made against the loop between domains II and III as well as three different antibodies raised against other various parts of the C-terminus of α _1_1.2 ( Davare *et al.*, 2000; Hell *et al.*, 1993a; Hell *et al.*, 1993b) ( Figure 1). FP1, CNC1, and the commercial antibody ACC-003 were raised against peptides covering middle portions of the II/III loop of α _1_1.2. The anti-phospho-S1700 antibody (pS1700) was produced against the respective phosphopeptide covering residues 1694-1713 in the C-terminus, the anti-phospho-S1928 antibody (pS1928) against the respective phosphopeptide covering residues 1923-1932, and CNC2 against residues 2122-2138 near the very C-terminus of α _1_1.2 ( Figure 1).

**Figure 2.**
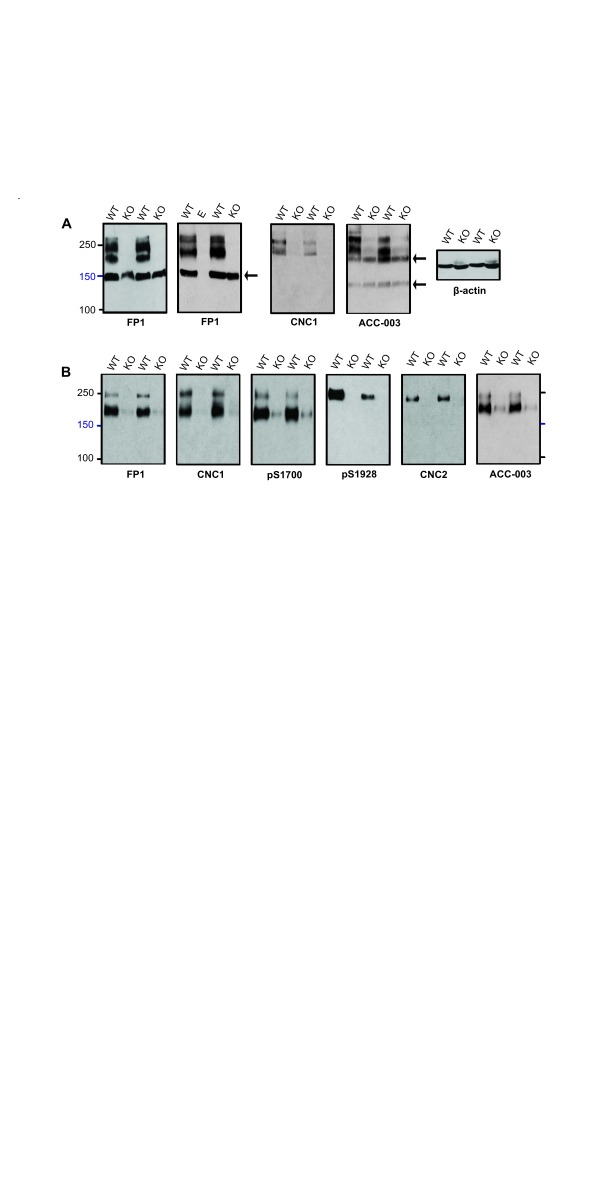
Determination of antibody specificity for α _1_1.2 with conditional α _1_1.2 KO mice. ( **A**) Immunoblots of Triton X-100 extracts from conditional α _1_1.2 KO mice (KO) and litter matched WT mice using gels polymerized from 8% acrylamide. To ensure that there was no spill-over between lanes, in some gels one or more lanes were left empty as shown here for the middle lane labeled E in the right FP1 blot. To fully resolve α _1_1.2 short and long forms, the 100 kDa marker was run close to the bottom except in the right panel. In this experiment, electrophoresis of the same extracts used for α _1_1.2 immunoblotting was terminated before the dye front reached the bottom. Probing for β-actin showed that comparable amounts of protein were present in each extract from the different WT and KI mice. ( **B**) Ca _v_1.2 was immunoprecipitated from brain extracts from conditional KO and WT mice with the FP1 antibody before SDS-PAGE in gels polymerized from 6% acrylamide and immunoblotting with the indicated antibodies. To fully separate α _1_1.2 short and long forms, electrophoresis was performed until the 100 kDa marker was near the bottoms of the gels. For all antibodies, the ~210 and 250 kDa bands were nearly or completely absent in cKO samples.

We tested whether immunoreactive bands recognized by these antibodies correspond to α _1_1.2 size forms using brain extracts from WT and α _1_1.2 KO mice. Total KO of α _1_1.2 is embryonically lethal due to the central role of Ca _v_1.2 triggering heart beat ( Seisenberger *et al.*, 2000). Thus we used tissue from conditional α _1_1.2 KO mice (cKO) in which the floxed α _1_1.2 gene was excised by Cre recombinase, whose expression was driven by the synapsin I promoter, resulting in a pan neuronal deletion throughout the brain ( Cui *et al.*, 2008; Zhu *et al.*, 2001). We extracted whole mouse brain with 1% Triton X-100 (solubilizing >90% of total Ca _v_1.2) and used the extracts directly for immunoblotting ( Figure 2A). FP1 detected clear, strong bands of apparent M _R_ of ~150, 210, and 250 kDa in WT mice. As expected for antibodies with immunoreactivity to α _1_1.2, these 210 and 250 kDa bands were not readily detectable when cKO brain tissue was probed with FP1. Accordingly, these bands constitute bona fide α _1_1.2 size forms. In contrast, the 150 kDa band was not only prominent in WT samples but also highly expressed in cKO brain, suggesting that this band does not correspond to α _1_1.2 sequences. This conclusion is further supported when similar blots were probed with CNC1, which only recognized bands of 210 and 250 kDa in WT brain, both of which were undetectable in immunoblot lanes containing lysate from cKO mice. The ACC-003 antibody, a commercial antibody designed against the same epitope, recognized similar 210 and 250 KDa bands present in WT but not cKO brains, which is again consistent with these bands representing true major α _1_1.2 size forms. However, this antibody detected additional immunoreactive bands of ~130 and ~190 kDa that were of equal strength in brain lysates from both WT and cKO mice, indicating that these two bands are not true isoforms of α _1_1.2.

For increased sensitivity and to further define the identity of the 150 kDa band detected in FP1 blots and the 130 and 190 kDa bands recognized by ACC-003, we performed immunoprecipitation to concentrate the α _1_1.2 isoforms from a much larger volume of lysate. The FP1 antibody (of which we have a significantly larger supply than of the other antibodies) was used to immunoprecipitate α _1_1.2 from Triton X-100 brain extracts. The resulting concentrate was then subjected to individual immunoblot analysis using the six distinct α _1_1.2 antibodies available. Remarkably, probing with FP1 only revealed a 210 and a 250 kDa band but not the 150 kDa band ( Figure 2B). Apparently this 150 kDa band detected by FP1 immunoblot of directly loaded brain extracts is not readily immunoprecipitated by FP1. This observation further suggests that the 210 and 250 kDa bands are immunologically different from the 150 kDa band, with the 210 and 250 kDa proteins but not the 150 kDa protein being efficiently immunoprecipitated. Moreover as with FP1, the CNC1, ACC-003, and pS1700 antibodies all recognized bands of 210 and 250 kDa in FP1 WT brain immunoprecipitates, whereas the more C-terminal directed pS1928 and CNC2 antibodies recognized only a single band of 250 kDa ( Figure 2B). FP1, CNC1, ACC-003, and pS1700 immunoblotting did, as expected, reveal faintly reactive 210 and 250 kDa bands after FP1 immunoprecipitation from cKO brains. These weakly immunoreactive bands are the result of the continued α _1_1.2 expression in non-neuronal tissue (glia and vasculature). Importantly, the 130 and 190 kDa bands recognized by ACC-003 in brain lysate of WT and cKO mice were not detectable after the FP1 immunoprecipitation. Similar to our observation that the 150 kDa band detected by FP1 probings of directly loaded brain lysates is not detected in blots of FP1 immunoprecipitates, this finding further indicates that the 130 and 190 kDa bands are not α _1_1.2 isoforms.

### The two prevalent size forms of α _1_1.2 are about 250 and 210 kDa

**Figure 3.**
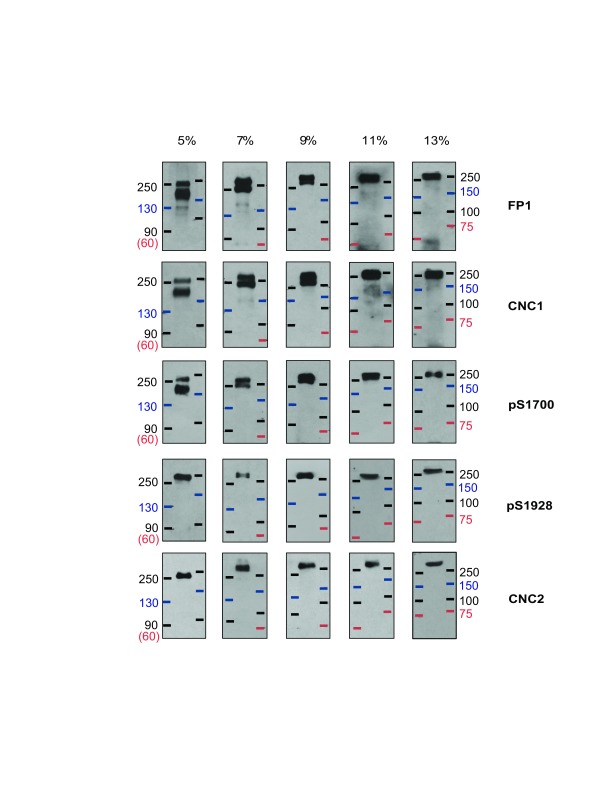
Analysis of α _1_1.2 size forms by SDS-PAGE with increasing acrylamide concentrations. Ca _v_1.2 was immunoprecipitated from mouse brain extracts (Triton X-100) with the FP1 antibody against α _1_1.2 before fractionation by SDS-PAGE in gels polymerized from 5, 7, 9, 11, and 13% acrylamide followed by immunoblotting with the indicated antibodies. Two different prestained marker protein sets were used to estimate M _R_.

Not all proteins, including M _R_ markers, consistently migrate at the same apparent molecular mass during SDS-PAGE. It is conceivable that a protein of a true M _R_ of 150 kDa could run with an apparent M _R_ of 200 kDa and more. To increase certainty about the M _R_ of the apparent 210 and 250 kDa bands detected in the above experiments and scrutinize whether the apparent 210 kDa band might under different conditions migrate near a 150 kDa marker, α _1_1.2 migration relative to two different M _R_ marker sets was analyzed in gels made from different concentrations of acrylamide (5–11%). For this analysis, Ca _v_1.2 was enriched by immunoprecipitation with FP1. The individual marker proteins in the two different M _R_ marker sets migrated uniformly and as expected for their molecular mass. Here, all five of the tested α _1_1.2 antibodies recognized a protein band that migrated with the 250 kDa size markers in 5% gels and slightly slower than the 250 kDa markers in all other % acrylamide gels ( Figure 3). The two loop antibodies FP1 and CNC1, as well as pS1700, but not pS1928 nor CNC2, recognized a second band that migrated either between the 150 and 250 kDa markers in 5% acrylamide gels or just below the 250 kDa markers in 7% gels, or co-migrated with the larger size form in 9, 11, and 13% gels. The pS1928 and CNC2 antibodies only detected the long form in brain extracts while the pS1700 antibody recognized both size forms, a pattern indicating that the shorter form represents an α _1_1.2 size variant that is truncated, relative to full length, between residues 1700 and 1928. This notion is consistent with a size difference between the long and short forms of roughly 30–60 kDa and is also in agreement with the observed migration for the lower FP1-, CNC1-, and pS1700 immunoreactive band in 5–7% gels (the phospho-serine 1700 being 471 residues upstream of the distal C-terminus of full length α _1_1.2).

In some cases, a faint immunoreactive band with an apparent M _R_ of ~130 kDa in 5% gels and ~150 kDa in 7%, 11% and 13% gels was observed by immunoblotting with CNC1 and FP1. Figure 3 shows the clearest examples among all our immunoblots for detection of this weak band by CNC1 and FP1. However, in the majority of experiments a similar sized band was not detectable.

**Figure 4.**
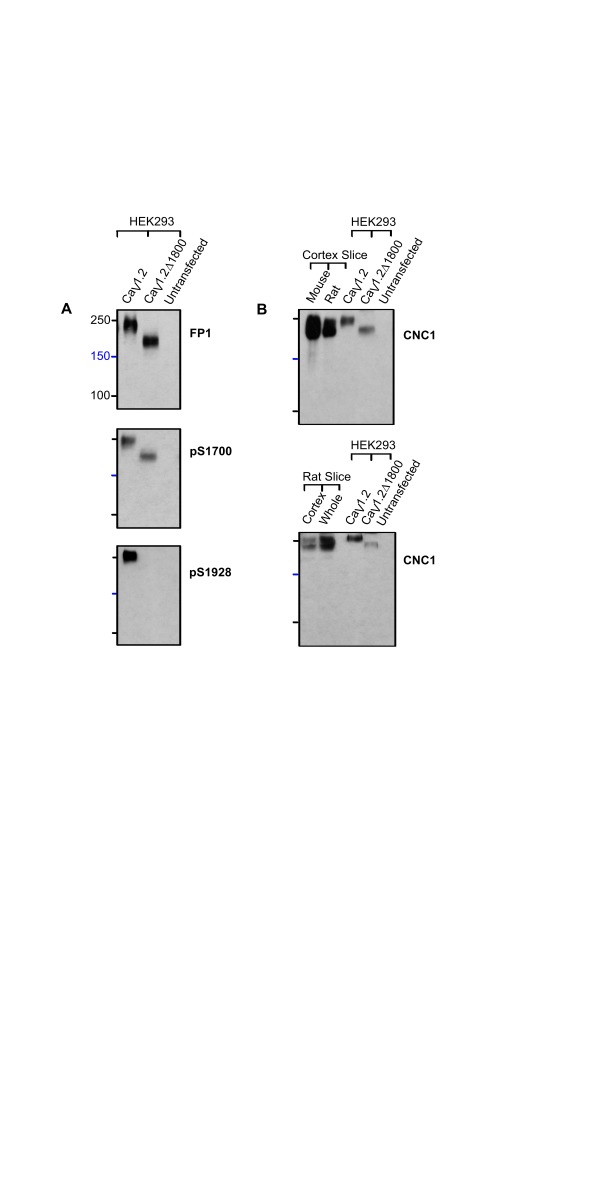
Mouse and rat α _1_1.2 short forms co-migrate with α _1_1.2 truncated at residue 1800 in the middle of the c-terminus. HEK293T cells were transfected with full length or truncated (Δ1800) α _1_1.2 plus α _2_δ _1_ and β _2a_. HEK293T cells and rat and mouse brain slices were extracted with 1% Triton X-100 before immunoprecipitation of α _1_1.2, SDS-PAGE in gels polymerized from 8% acrylamide, and immunoblotting with the indicated antibodies. ( **A**) The full length form of α _1_1.2 expressed in HEK293 cells migrated with an apparent M _R_ of 250 kDa and is detected by FP1, pS1700 and pS1928. Truncated Δ1800 α _1_1.2 migrated with an apparent M _R_ of 210 kDa and is detected by FP1 and pS1700 but not pS1928. ( **B**) The α _1_1.2 short and long form appear only partially resolved because the weak α _1_1.2 signals in HEK293 cell samples required long exposure times. The upper band as detected by CNC1 after FP1 immunoprecipitation from rat and mouse forebrain slices and cortical slices co-migrated with the full length form of α _1_1.2 expressed in HEK293 cells, while the lower band co-migrated with the truncated Δ1800 α _1_1.2 expressed in HEK293 cells.

Given the anomalous migration of the short form, we wanted to provide further evidence for the estimation of a 30-60 kDa difference between the two size forms. α _1_1.2 was expressed in HEK293 cells as either its full length form or as a shortened version truncated at residues 1800 (α _1_1.2Δ1800) before extraction, immunoprecipitation and separation by 7% SDS-PAGE. As with the mouse brain lysate samples, FP1 and pS1700 detected full length and truncated α _1_1.2 with an apparent M _R_ of about 250 and 210 kDa, respectively, whereas the pS1928 antibody only identified the full length α _1_1.2 ( Figure 4A).

Additional experiments were performed with rat tissue to look for potential differences in proteolytic processing between mouse and rat α _1_1.2. We extracted forebrain slices and cortical slices from both mouse and rat for immunoprecipitation with FP1 and separation by SDS-PAGE, matching the 8% acrylamide gel conditions used in ( Michailidis *et al.*, 2014). As expected from our earlier analysis in 7 and 9% acrylamide gels, the α _1_1.2 short form was partially separated from the long form in the 8% acrylamide gel ( Figure 4B). Importantly, the long and short forms from the rodent brain tissues co-migrated with the corresponding full length α _1_1.2 and α _1_1.2Δ1800 ectopically expressed in HEK293 cells. Accordingly, truncation of the long form at approximately residue 1800 is most likely what gives rise to the main α _1_1.2 short form in rodent brain. Moreover, these experiments did not reveal a protein band isolated from rat brain lysates that could conceivably correspond to a 150 kDa size form of α _1_1.2, and only a very weak band of ~150 kDa could be detected in the mouse samples.

**Figure 5.**
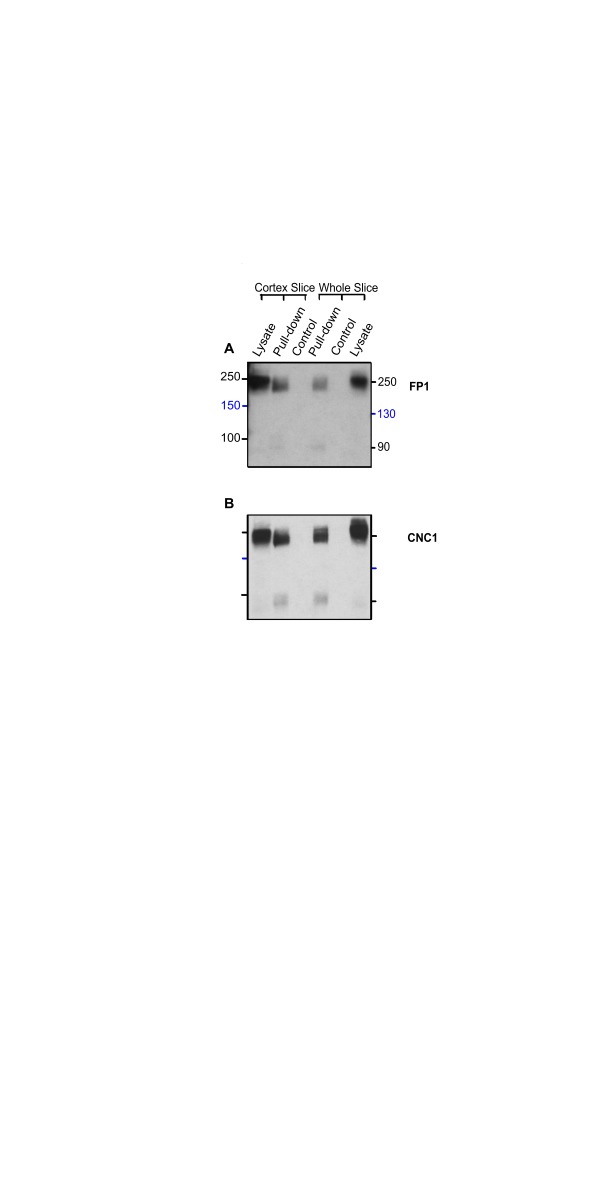
Surface biotinylation labels α _1_1.2 size forms with apparent M _R_ > 200 kDa in rat cortical and forebrain slices. Cortical and forebrain slices were surface biotinylated and solubilized before pulldown with NeutrAvidin Sepharose, SDS-PAGE in 8% acrylamide gels, and immunoblotting with CNC1 and FP1. Control reflects slices mock treated without Sulfo-NHS-SS-biotin to demonstrate specificity of pulldown. Twenty μL lysate was also directly loaded for comparison.

To test whether pull-down of surface biotinylated proteins might enrich for a unique α _1_1.2 population at the cell surface and thereby unmask a size form smaller than 200 kDa, we performed surface biotinylation of acute slices using acute slices made from both total rat brain and cortex before extraction. We then carried out neutravidin-Sepharose pulldown and immunoblotting as described earlier ( Michailidis *et al.*, 2014). In agreement with our findings above, CNC1 and FP1 immunoblotting of proteins in neutravidin-Sepharose pulldowns and total lysate loads separated by 8% PAGE revealed major partially separated bands at ~200–250 kDa and no evidence of a 150 kDa band ( Figure 5). On some immunoblots a weak band within the 90 kDa range was detectable by CNC1 ( Figure 5B). Neutravidin pulldowns of unbiotinylated control samples did not yield detectable immunoblot signals, verifying the specificity of the biotin-neutravidin pulldown assay.

### The strong 150 kDa band detected with FP1 in lysate is different from the faint 150 kDa band detected after FP1 immunoprecipitation

**Figure 6.**
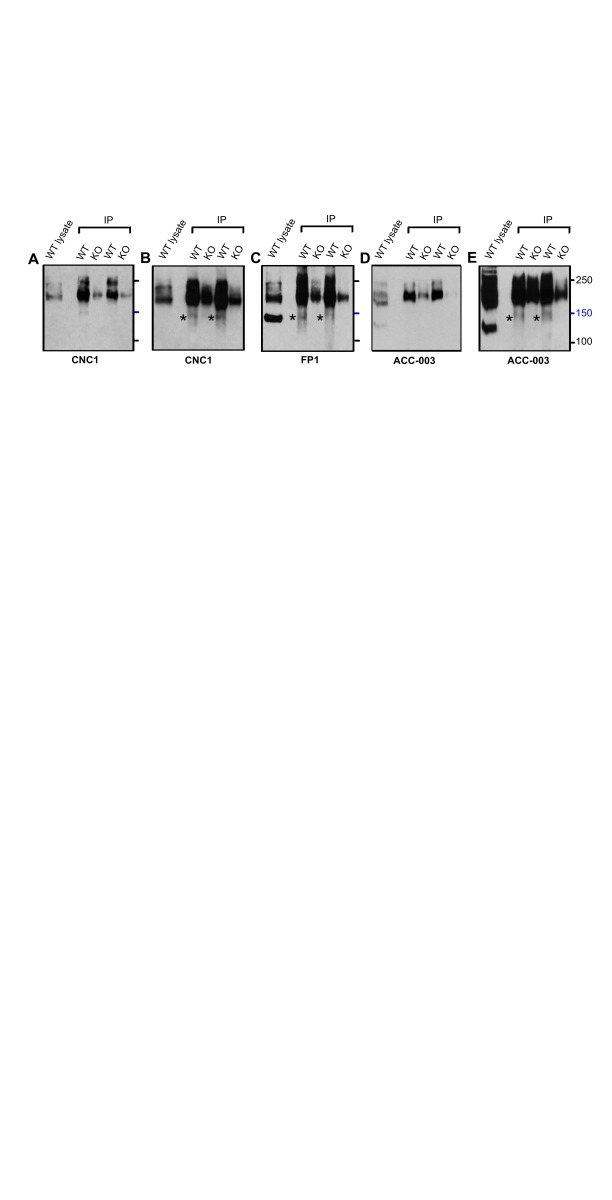
Differential recognition of the strong 150 kDa FP1 band in lysate and weak 150 kDa band by FP1, CNC1, and ACC-003 after IP of α _1_1.2 with FP1. Immunoblots with CNC1 ( **A**, **B**), FP1 ( **C**), and ACC-003 ( **D**, **E**) of Triton X-100 extracts from WT mice (lysate) and after immunoprecipitation with FP1 from cKO and WT mice. Gels were polymerized from 8% acrylamide. Note that a weak 150 kDa band is detected by CNC1, FP1, and ACC-003 after enrichment of α _1_1.2 by immunoprecipitation with FP1 but the strongly immunoreactive 150 kDa band detected by FP1 in lysate is not detectable by either CNC1 or ACC-003.

Because we observed in some experiments a weak ~150 kDa band in FP1 immunoprecipitates that were immunoblotted with FP1 and CNC1 ( Figure 3), we wanted to clarify whether this band is related to the strong 150 kDa band detected with FP1 in brain lysate of WT and cKO mice. We ran in parallel forebrain extracts and FP1 immunoprecipitates on the same gel ( Figure 6). As before ( Figure 2), CNC1 did not detect a 150 kDa band in lysate lanes ( Figure 6A) even when blots were exposed to film for longer time periods ( Figure 6B). However upon prolonged film exposure CNC1 probed blots reveal a faint 150 kDa band in lanes for the FP1 immunoprecipitated samples isolated from WT mice. Extended film exposure also revealed a weak 150 kDa band detected by FP1 after immunoprecipitation with FP1 ( Figure 6C). Because the faint band in FP1 immunoprecipitates is equally well detected by FP1 and CNC1 but the strong 150 kDa band seen with FP1 in lysate is only detected by FP1, the two ~ 150kDa bands are most likely not related to one another but rather represent different protein species. If these ~150kDa bands were the same protein the CNC1 antibody should detect the strong 150 kDa band in lysate as well. Finally, only a faint 150 kDa band was also detected by the ACC-003 antibody probe upon extended exposure of the blot to film ( Figure 6E).

Raw data supporting the findings presented in this study The raw data shows full size film images of probed membranes. Full size membranes resulting from transfer of full size gels were often vertically cut to separate replicate sets of samples typically separated by M _R_ markers for simultaneous probing of the different membrane fragments with different antibodies. For optimal resolution of the α _1_1.2 long and short forms, which exhibit high M _R_, gels were run until the 60 kDa M _R_ marker was either close to the very bottom of the gel or completely run off.
 
**Raw data for Figure 2. Determination of antibody specificity for α _1_1.2 with conditional α _1_1.2 KO mice.**
Original source images for Figure 2:
(A) Immunoblots of Triton X-100 extracts from conditional α _1_1.2 KO mice (KO) and litter matched WT mice using gels polymerized from 8% acrylamide. To ensure that there was no spill-over between lanes, in some gels one or more lanes were left empty as shown here for the middle lane labeled E in the right FP1 blot. To fully resolve α _1_1.2 short and long forms, the 100 kDa marker was run close to the bottom except in the right panel. In this experiment, electrophoresis of the same extracts used for α _1_1.2 immunoblotting was terminated before the dye front reached the bottom. Probing for b-actin showed that comparable amounts of protein were present in each extract from the different WT and KI mice.
(B) Ca _v_1.2 was immunoprecipitated from brain extracts from conditional KO and WT mice with the FP1 antibody before SDS-PAGE in gels polymerized from 6% acrylamide and immunoblotting with the indicated antibodies. To fully separate α _1_1.2 short and long forms, electrophoresis was performed until the 100 kDa marker was near the bottoms of the gels. For all antibodies, the ~210 and 250 kDa bands were nearly or completely absent in cKO samples.
 
**Raw data for Figure 3. Analysis of α _1_1.2 size forms by SDS-PAGE with increasing acrylamide concentrations.**
Original source images for Figure 3: Ca _v_1.2 was immunoprecipitated from mouse brain extracts (Triton X-100) with the FP1 antibody against α _1_1.2 before fractionation by SDS-PAGE in gels polymerized from 5, 7, 9, 11, and 13% acrylamide followed by immunoblotting with the indicated antibodies. Two different prestained marker protein sets were used to estimate M _R_.
 
**Raw data for Figure 4. Mouse and rat α _1_1.2 short forms co-migrate with α _1_1.2 truncated at residue 1800 in the middle of the c-terminus.**
Original source images for Figure 4: HEK293T cells were transfected with full length or truncated (Δ1800) a _1_1.2 plus a _2_d _1_ and b _2a_. HEK293T cells and rat and mouse brain slices were extracted with 1% Triton X-100 before immunoprecipitation of a _1_1.2, SDS-PAGE in gels polymerized from 8% acrylamide, and immunoblotting with the indicated antibodies.
(A) The full length form of a _1_1.2 expressed in HEK293 cells migrated with an apparent M _R_ of 250 kDa and is detected by FP1, pS1700 and pS1928. Truncated D1800 a _1_1.2 migrated with an apparent M _R_ of 210 kDa and is detected by FP1 and pS1700 but not pS1928.
(B) The a _1_1.2 short and long form appear only partially resolved because the weak a _1_1.2 signals in HEK293 cell samples required long exposure times. The upper band as detected by CNC1 after FP1 immunoprecipitation from rat and mouse forebrain slices and cortical slices co-migrated with the full length form of a _1_1.2 expressed in HEK293 cells, while the lower band co-migrated with the truncated D1800 a _1_1.2 expressed in HEK293 cells. Sometimes, as seen here, a significant portion of the pore-forming subunit aggregated at the interface between stacking and resolving gels. This unresolved fraction (thick arrow) is not representative of its true molecular mass and not shown in the main figures.
 
**Raw data for Figure 5. Surface biotinylation labels α _1_1.2 size forms with apparent M _R_ > 200 kDa in rat cortical and forebrain slices.**
Original source images for Figure 5: Cortical and forebrain slices were surface biotinylated and solubilized before pulldown with NeutrAvidin Sepharose, SDS-PAGE in 8% acrylamide gels, and immunoblotting with CNC1 and FP1. Control reflects slices mock treated without Sulfo-NHS-SS-biotin to demonstrate specificity of pulldown. Twenty mL lysate was also directly loaded for comparison.
 
**Raw data for Figure 6. Differential recognition of the strong 150 kDa FP1 band in lysate and weak 150 kDa band by FP1, CNC1, and ACC-003 after IP of α _1_1.2 with FP1.**
Original source images for Figure 6: Immunoblots with CNC1 (A,B), FP1 (C), and ACC-003 (D,E) of Triton X-100 extracts from WT mice (lysate) and after immunoprecipitation with FP1 from cKO and WT mice. Gels were polymerized from 8% acrylamide. Note that a weak 150 kDa band is detected by CNC1, FP1, and ACC-003 after enrichment of α _1_1.2 by immunoprecipitation with FP1 but the strongly immunoreactive 150 kDa band detected by FP1 in lysate is not detectable by either CNC1 or ACC-003. Click here for additional data file. Copyright: © 2018 Buonarati OR et al. 2018 Data associated with the article are available under the terms of the Creative Commons Zero "No rights reserved" data waiver (CC0 1.0 Public domain dedication).

**Figure 7.**
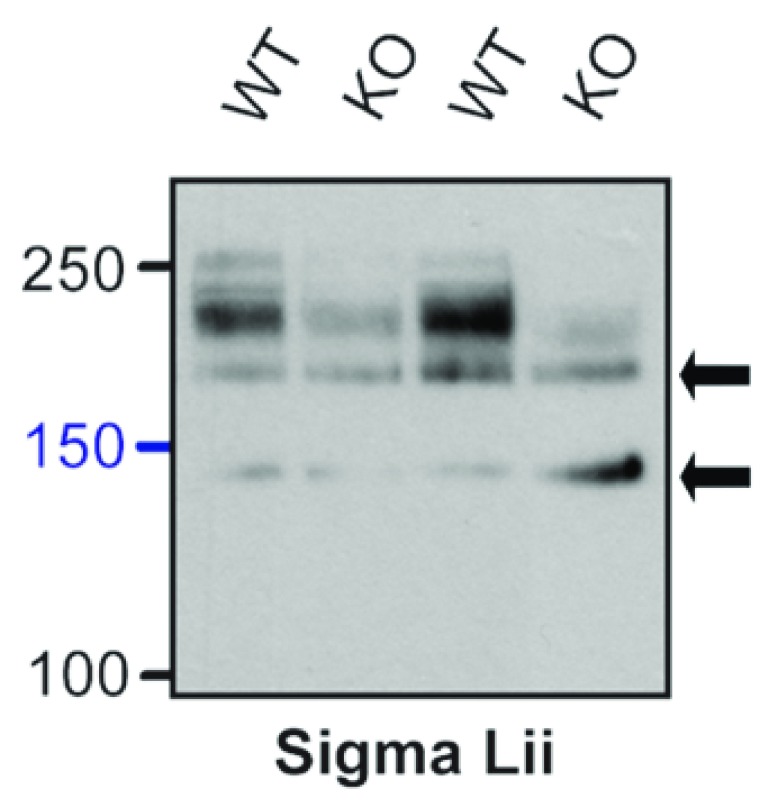
Determination of Sigma Lii Specificity for α _1_1.2 with Conditional α _1_1.2 KO Mice. Sigma Lii immunoblots of Triton X-100 extracts from cKO and WT mice using gels polymerized from 8% acrylamide. To fully resolve α _1_1.2 short and long forms, electrophoresis was performed until the 100 kDa marker was near the bottoms of the gels. As with other antibodies tested (see Figure 2), the ~210 and 250 kDa bands were nearly or completely absent in cKO samples. Arrows point to bands at 130 and 180 kDa that are present in WT and cKO tissue.

To provide further characterization of antibodies against Ca _v_1.2, we obtained the Sigma Lii antibody as used by Michailidis *et al.* (2014), which, according to personal communication with a Sigma representative, is actually sourced from Alomone. We tested its specificity on tissue from conditional Ca _v_1.2 knock-out mice, as in Figure 2. The immunoblot images look very similar to those for ACC-003 with immunoreactive bands around 210 and 250 kDa that are strongly reduced in the conditional Ca _v_1.2 KO mice and bands around 130 and 180 kDa that are undiminished in the KO tissue ( Figure 7). Apparently, like ACC-003, Sigma Lii recognizes two nonspecific bands that are not related to Ca _v_1.2 at ~130 and ~180 kDa.

**Figure 8.**
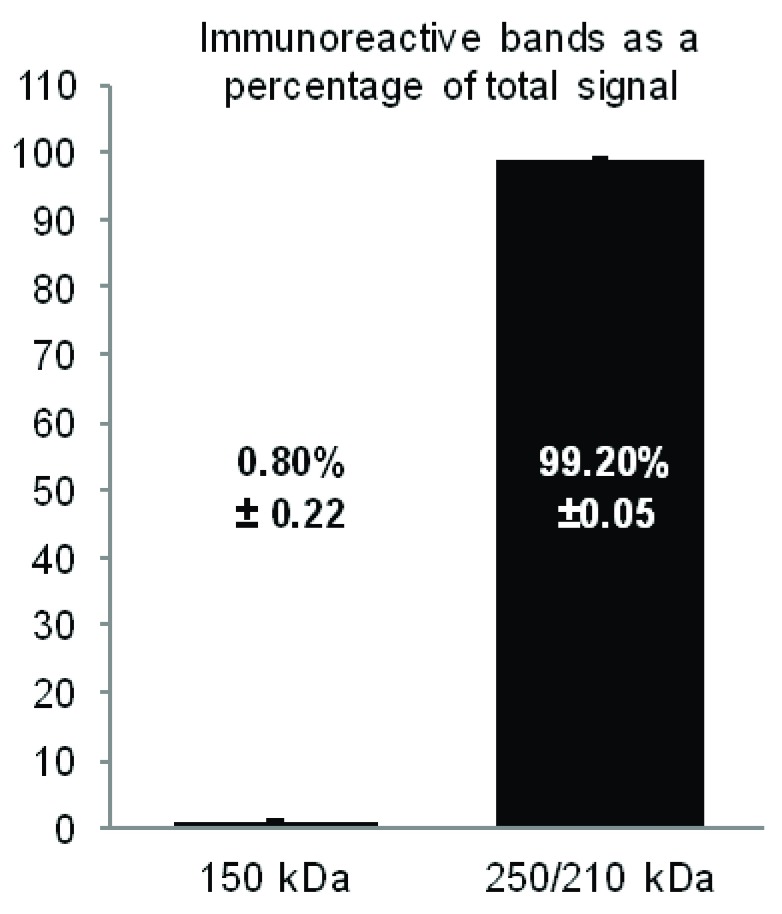
Quantification of α _1_1.2 Size Forms. Ca _v_1.2 was immunoprecipitated from mouse brain extracts (Triton X-100) with FP1 before fractionation by SDS-PAGE and immunoblotting with FP1 and CNC1 antibodies (see Figure 3 and Figure 6). All α _1_1.2 immunoreactive bands at the longest film exposure were quantified in Adobe Photoshop, shown here as average percent of total immunoreactivity per blot.

We quantified relative signal intensity for the faint 150 kDa band in Figure 3 and Figure 6 that is detectable after immunoprecipitation with FP1 by both CNC1 and FP1. Accordingly, the band intensity at 150 kDa is 0.8 ± 0.22% of total α _1_1.2 intensity, compared to 99.2 ± 0.05% for the 210 and 250 kDa bands combined ( Figure 8). This finding suggests once more that the α _1_1.2 subunit does exist in a 150 kDa fragment as reported by Michailidis *et al.* (2014) but this size form only constitutes a minuscule fraction the α _1_1.2 population.

**Figure 9.**
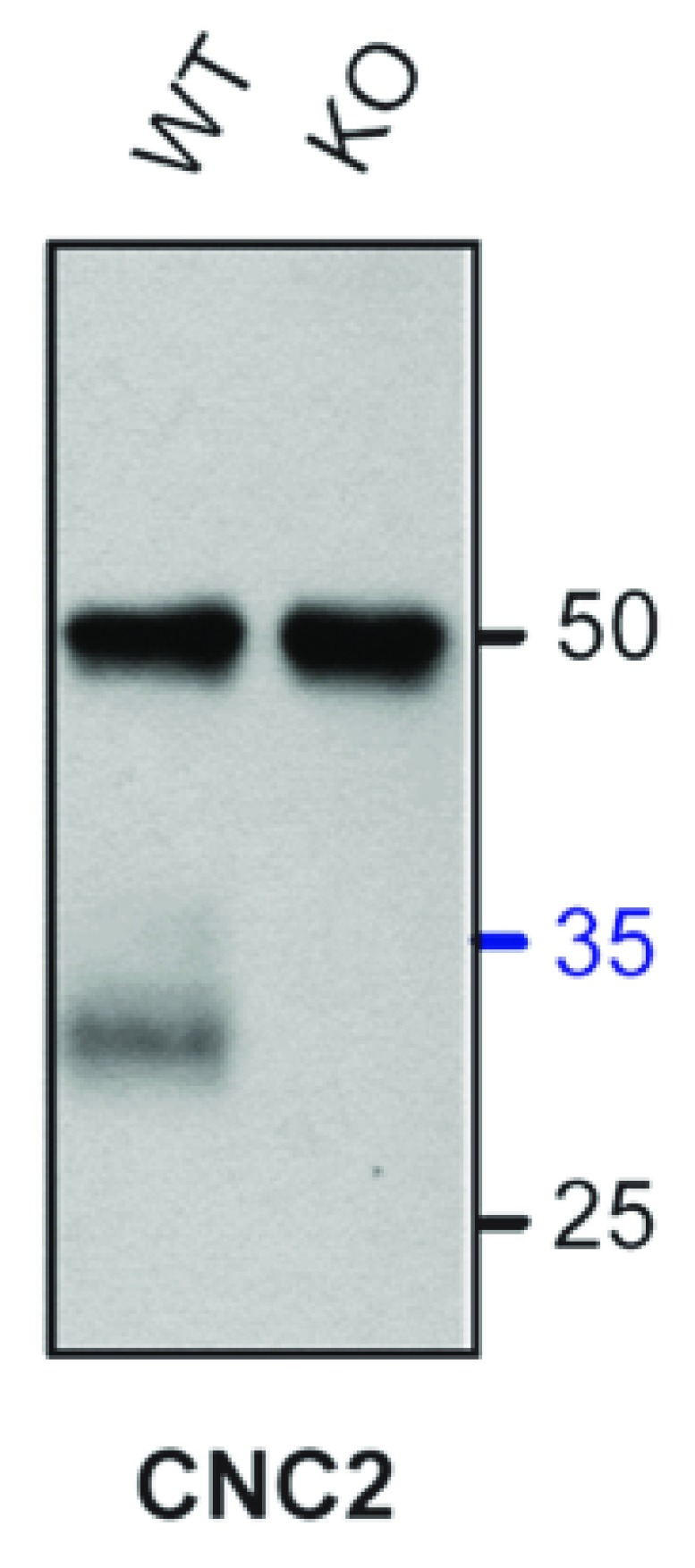
Detection of α _1_1.2 distal C-terminus. CNC2 immunoblots showed an immunoreactive band migrating near 30 kDa in Triton X-100 forebrain extracts after immunoprecipitation with FP1. Signal was absent in immunoprecipitates from cKO samples. Gels were polymerized from 13% acrylamide. Note that this interaction was variable, with roughly half of 8 IPs tested showing a clear, specific band, perhaps because this fragment is unstable and proteolytically degraded. Also, the 50 kDa bands are most likely the heavy chains of the immunoprecipitating antibody.

We also ran higher acrylamide percentage gels (13%) to resolve and detect the C-terminal fragment that is expected to result from cleavage of α _1_1.2 in the middle of its C-terminus. Probing with our CNC2 antibody (raised against a segment in the distal C-terminus) does indeed detect a band of ~30 kDa, the predicted molecular mass of the short cleavage product ( Figure 9). This is consistent with earlier reports ( Fuller *et al.*, 2010; Gao *et al.*, 2001; Hulme *et al.*, 2006b) indicating that the C-terminal fragment produced by α _1_1.2 proteolysis stays physically and functionally associated with the cleaved channel.

**Figure 10.**
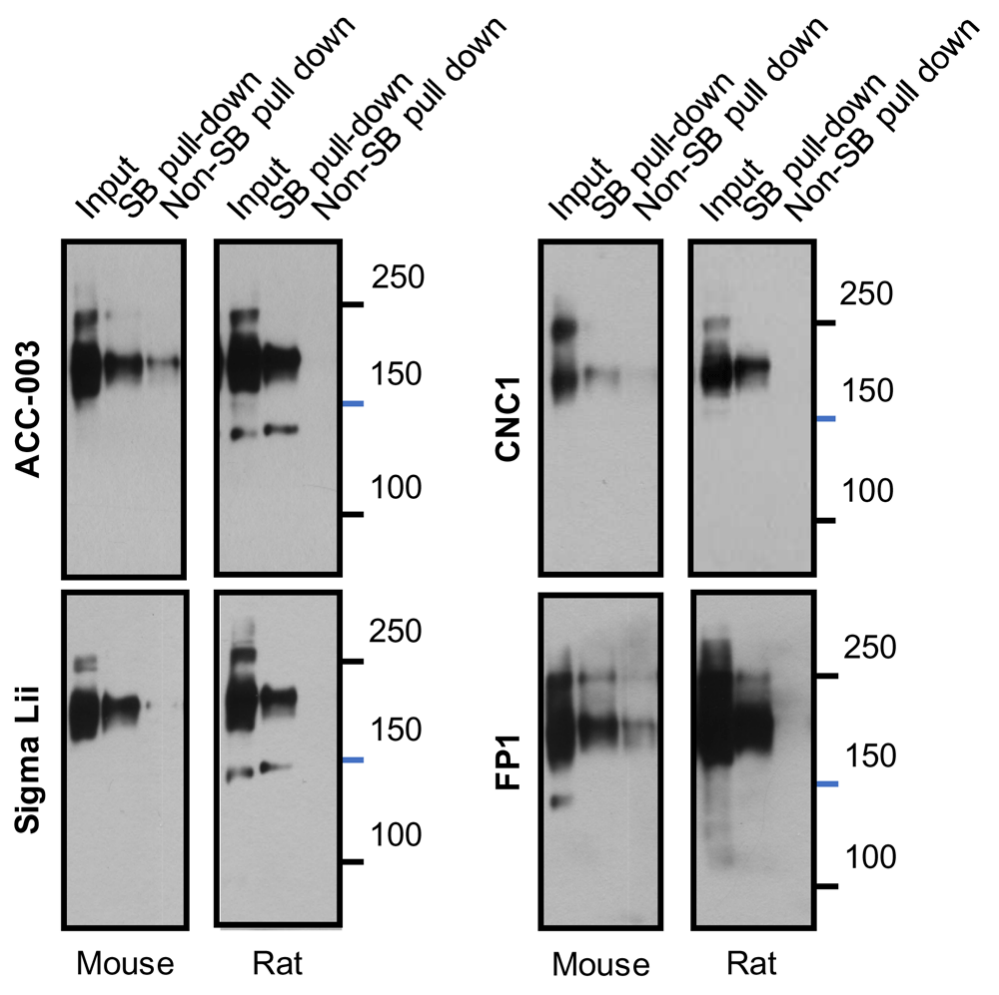
Differential Recognition of a 150 kDa Band in Surface Biotinylated Rat and Mouse Cortical Slices. Acute cortical slices were surface biotinylated and solubilized before pull down with NeutrAvidin Sepharose, SDS-PAGE in 8% acrylamide gels, and immunoblotting with CNC1, FP1, ACC-003, and Sigma Lii. Control reflects slices mock treated without Sulfo-NHS-SS-biotin to demonstrate specificity of pulldown. Twenty μL lysate was also directly loaded for comparison.

We repeated surface biotinylation as performed by Michailidis *et al.* (2014) of acute cortical slices before pull down with streptavidin agarose, in an additional attempt to detect the 150 kDa fragment described by these authors ( Figure 10). Accordingly, both ACC-003 and Sigma Lii detected a band of ~150 kDa in rat but interestingly not mouse tissue. Importantly, neither CNC1 nor FP1 detected such a 150 kDa band following surface biotinylation even though signals of the upper two bands are of similar intensity as for ACC-003 and Sigma Lii. It is theoretically possible that CNC1, which was made against a fairly small segment of the loop between domains II and III (residues 818-835; Figure 1), does not detect the 150 kDa fragment because cleavage leading to this fragment would occur right within the epitope region. However, the finding that FP1, which was made against a much larger fragment of loop II/III (residues 783-845), doesn’t detect a 150 kDa band either argues that the 150 kDa band is not of α _1_1.2 origin. The non-specific 150 kDa band that had been detected by FP1 in mouse brain lysate ( Figure 2A) is clearly visible again in the input lane but undetectable in the fraction of surface biotinylated Ca _v_1.2 ( Figure 10). Such lack of detectability in the surface biotinylated Ca _v_1.2 fraction argues once more against the idea that the 150 kDa band seen in lysate reflects an α _1_1.2 fragment that would specifically be localized at the cell surface.

**Figure 11.**
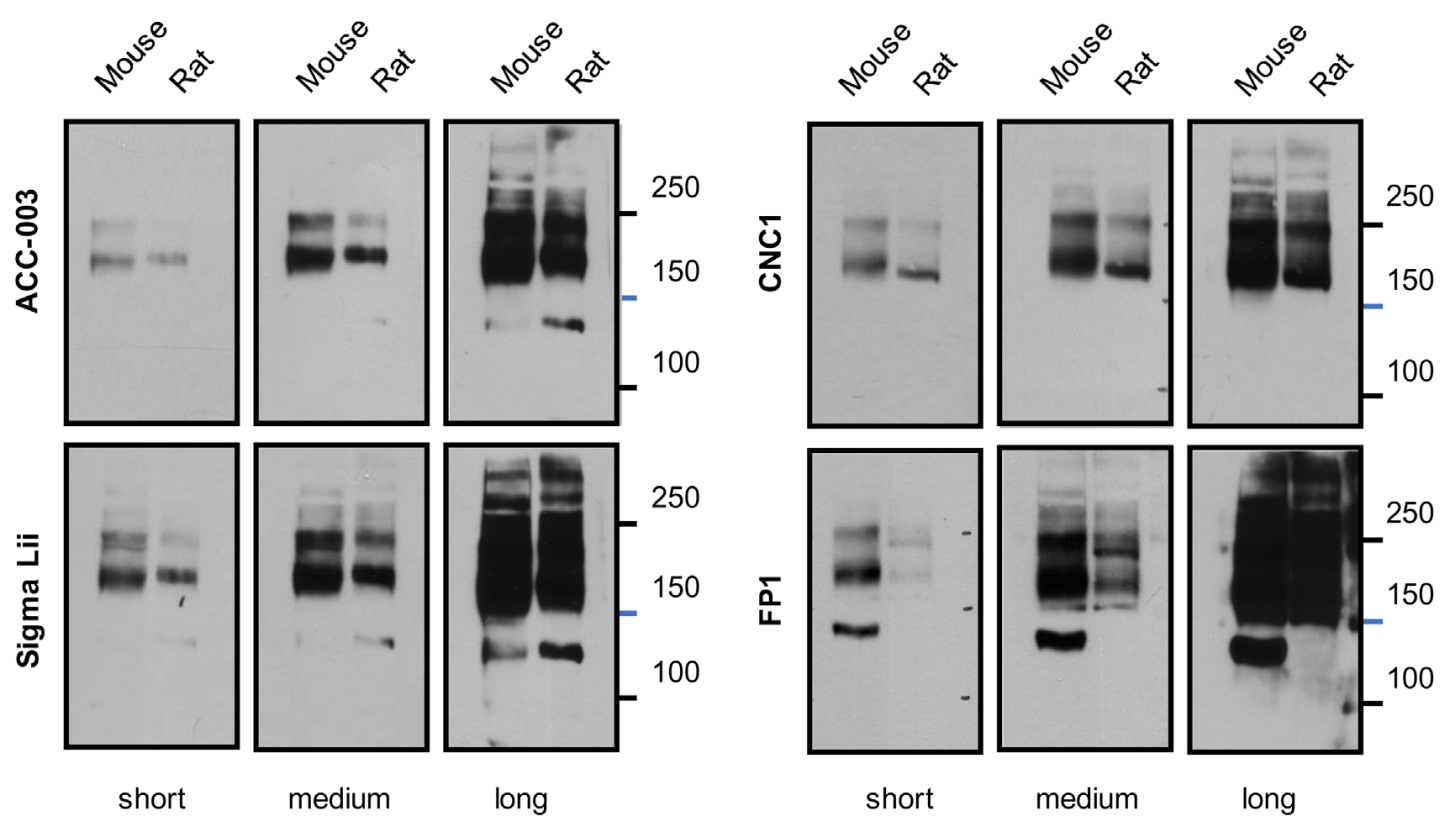
Comparison of α _1_1.2 Immunoreactivity in Rat and Mouse Lysate. Immunoblots with ACC-003, Sigma Lii, CNC1, and FP1 of Triton X-100 extracts from WT mice and rat forebrain. Gels were polymerized from 5% acrylamide and electrophoresis was performed until the 100 kDa marker was near the bottoms of the gels. Note that a weak 150 kDa band is detected by ACC-003 and Sigma Lii in both mouse and rat lysates. In comparison, a strongly immunoreactive 150 kDa band is detected by FP1 in mouse but not rat lysate.

To further characterize this 150 kDa band detected by FP1 we ran additional sets of gels polymerized from 5% acrylamide to detect α _1_1.2 in mouse and rat forebrain lysate. Again, FP1 recognized a quite prominent ~150 kDa band in mouse but not rat lysate ( Figure 11). ACC-003 and Sigma Lii recognized a band of similar size in both mouse and rat lysates.

Given that a prominent 150 kDa band analogous to the one described by Michailidis *et al.* (2014) is not detectable in lysate by CNC1, surface biotinylated Ca _v_1.2 fractions, and FP1 immunuoprecipitates (except for a very faint band <1% of total signal) and that FP1 doesn’t detect a 150 kDa band in surface biotinylated Ca _v_1.2 fractions, we propose that the 150 kDa band by Michailidis *et al.* (2014) largely reflects a non-specific species except perhaps for a very small amount of midchannel cleaved α _1_1.2 subunit. Where this tiny Ca _v_1.2 fraction might reside and have functional relevance remains to be determined.

## Discussion

Our extensive and detailed biochemical analysis of α _1_1.2 size forms was inspired by recent work that suggested that surface α _1_1.2 is cleaved to a large degree between domains II and III ( Michailidis *et al.*, 2014). The main evidence for the midchannel proteolysis proposed in this publication was based on immunoblotting:

1. The anti-L _II-III_ antibody (ACC-003 from Alomone), detected two main bands that migrated with apparent M _R_ values of ~150 and ~250 kDa (like CNC1, ACC-003 was made against α _1_1.2 residues 818-835 in the loop between domains II and III);
2. An antibody produced against residues 2127-2143 near the very C-terminus of α _1_1.2 (anti-L _Ct_) recognized ~9 bands of varying intensities, one of which exhibited intermediate labeling intensity at an apparent M _R_ of ~150 kDa;
3. An antibody against the N-terminus of α _1_1.2 (anti-L _Nt_) detected ~8 bands of varying intensities, including one of strong intensity that migrated with an apparent M _R_ of ~90 kDa.

These observations are consistent with the possibility that cleavage could occur just N-terminal to the recognition site of ACC-003 / anti-L _II-III_ inside the loop between domains II and III ( Michailidis *et al.*, 2014). The 250 kDa fragment recognized by ACC-003 / anti-L _II-III_ would reflect the full length channel and the 150 kDa fragment recognized by ACC-003 / anti-L _II-III_ would represent a fragment that comprises most of loop II/III, domains III and IV, and the full length C-terminus. The 90 kDa band detected with the N-terminal antibody would be the other cleavage product of the proposed midchannel cleavage and the anti-L _Ct_ recognized 150 kDa band would be the remaining C-terminal cleavage product. However, it remains untested and unclear whether the N- and C-terminal antibodies in this work did indeed recognize their intended target and which among the many bands detected by these antibodies were truly α _1_1.2, and not cross reactive proteins. Moreover, the 150 kDa band recognized by the anti-L _Ct_ antibody was a minor fraction of all the many bands detected by the anti-L _Ct_ antibody whereas the 150 kDa band recognized by the ACC-003 / anti-L _II-III_ antibody was one of two major bands detected by the anti-L _II-III_ antibody, making it unlikely that those two 150 kDa bands originated from the same protein.

One potential explanation for the detection of an apparent 150 kDa form of α _1_1.2 ( Michailidis *et al.*, 2014) is that the full length α _1_1.2 form and the C-terminally truncated form that we identify as 210 kDa in size were well separated (as in our 5% gels) but the 150 kDa M _R_ marker ran slower in their experiments than expected, which is possible for pre-stained markers. It is also possible that the 210 kDa form ran faster than anticipated or that a combination of both occurred. These effects would result in an apparent M _R_ value of our 210 kDa band that is less than the actual M _R_. Consistent with this possibility, the N-terminal antibody used in the previous work ( Michailidis *et al.*, 2014) recognized in addition to the 90 kDa band a 150 kDa band, which could be an overly fast migrating 210 kDa polypeptide. Importantly, by demonstrating precise co-migration of the short form with α _1_1.2Δ1800 ectopically expressed in HEK293 cells, we ruled out the possibility that the short α _1_1.2 form we identified with an apparent M _R_ of ~210 kDa is actually a significantly smaller fragment (potentially with an M _R_ of 150 kDa) that ran slower than would be expected for a polypeptide with an M _R_ substantially below 210 kDa ( Figure 4). Thus, the short α _1_1.2 form we observed following isolation from rodent brains lacks ~371 C-terminal residues of full length α _1_1.2, as is the case for α _1_1.2Δ1800.

Based on our analysis of cKO brain extracts, the most likely explanation is that the earlier 150 kDa band detected by Michailidis *et al*. ( Michailidis *et al.*, 2014) was not a significant α _1_1.2 isoform but rather a different protein recognized by the ACC-003 / anti-L _II-III_ loop antibody. In fact, in addition to the 210 and 250 kDa bands seen only in α _1_1.2 WT tissue and thereby reflecting major α _1_1.2 size forms, the ACC-003 antibody we obtained from Alomone Labs did recognize an ~130 and an ~190 kDa band, which were present not only in α _1_1.2 WT but also cKO mice. Similarly, another recent report indicates that the ACC-003 used in that work detected a band of ~130 kDa that was equally present in α _1_1.2 WT and cKO tissue when the 250 kDa band was only present in WT but not cKO tissue ( Bavley *et al.*, 2017). It is unclear whether the 150 kDa band recognized by the ACC-003 / anti-L _II-III_ antibody in the earlier work ( Michailidis *et al.*, 2014) corresponds to the 130 kDa band we detect with the ACC-003 antibody. This explanation is quite conceivable as migration behavior of native proteins (and even M _R_ markers) can easily vary between gel systems, as we showed for the ~210 kDa α _1_1.2 size form in Figure 2 and discussed in the preceding paragraph. Alternatively, cross-reactivity of antibodies with proteins other than α _1_1.2 could be different for the ACC-003 / anti-L _II-III_ antibody batch used more than 2 years ago ( Michailidis *et al.*, 2014) and the ACC-003 antibody we received in 2016 from Alomone Labs. Such differences could be due to different immune system responses within the individual rabbits used for immunization at different times. This possibility would also explain why the ACC-003 antibody we obtained from Alomone Labs recognized a cross-reacting 190 kDa band when the earlier ACC-003 / anti-L _II-III_ antibody did not ( Michailidis *et al.*, 2014).

In further support of the notion that antibodies against peptides derived from the L _II-III_ loop of α _1_1.2 can cross react with other proteins, our FP1 antibody recognizes a 150 kDa band of equal strength in extracts from WT and cKO brains, whereas the 210 and 250 kDa bands are strong in WT extracts and very faint in cKO extracts, the latter reflecting α _1_1.2 expression in non-neuronal tissue and cells ( Figure 2A). FP1 was made against a polypeptide spanning residues 783-845, which includes all of the residues of the synthetic peptide used to make the ACC-003 / anti-L _II-III_ antibody, as well as our CNC1 antibody (residues 821-838). Perhaps, the 821-838 segment mimics not only the α _1_1.2 epitope but also to some degree, though not perfectly, a related epitope on another protein that is present in WT and cKO mice. In fact, another antiserum that was produced completely independent from our CNC1 antibody but utilized the very same α _1_1.2 peptide sequence also detected an ~150 kDa band of similar intensity in brain lysates from WT and cKO mice ( Tippens *et al.*, 2008). Of note, the cKO mice used by Tippens *et al*. are different from the cKO used by us indicating that the strong 150 kDa band is present in mice of several different genetic backgrounds. Concordant with this idea, neither the ACC-003 antibody that we received from Alomone Labs that recognized a 130 kDa band in WT and cKO brains nor our CNC1 antibody recognized the strong 150 kDa band seen with FP1 in brain lysate. This finding is, once more, likely due to variability in immune responses of the individual rabbits to the immunogen, which at times but not always gives rise to antibodies against this unknown 150 kDa protein.

The results of our rigorous testing and validation of the antibodies used herein (see also Hall *et al.*, 2013) boosts our confidence that the ~250 kDa protein detected by all six antibodies and the ~210 kDa protein detected by the four antibodies that recognize epitopes upstream of residue 1800 are two different size forms of α _1_1.2. In contrast, the ~130 and ~150 kDa bands detected with ACC-003 / anti-L _II-III_ and FP1, respectively, are most likely not related to α _1_1.2 as these bands persist in α _1_1.2 cKO tissue. Overall, the evidence is overwhelming that the prominent bands in the 130-150 kDa range detected by the various anti-α _1_1.2 antibodies represent proteins that are different from α _1_1.2.

Theoretically, it is also possible that the 150 kDa protein species arose because of nonspecific post mortem proteolysis. Because we use a strong and well-defined cocktail of inhibitors against serine, cysteine, and metalloproteases ( Hell *et al.*, 1993a; Hell *et al.*, 1993b; Westenbroek *et al.*, 1992) (see Material and Methods) and are particularly careful to keep all samples cold, such proteolysis may not have occurred to a significant degree in our hands. Accordingly, we only detected at best a very weak band migrating with an apparent M _R_ of 150 kDa with the four different antibodies that recognized also full length α _1_1.2. Under less stringent conditions, greater proteolysis might occur post mortem during tissue extraction, biotinylation, and purification. We tested whether incubation of forebrain slices at room temperature without O _2_ supply for 10 and 20 min would trigger proteolytic processing that results in a 150 kDa α _1_1.2 band. However, in several different experiments we did not observe any increase in the weak 150 kDa band that is detected by either FP1 or CNC1 after enrichment of Ca _v_1.2 by immunoprecipitation with FP1 (data not shown). Thus it appears unlikely that any 150 kDa band is due to post mortem proteolytic processing of α _1_1.2.

If the 150 kDa band reported in the previous work ( Michailidis *et al.*, 2014) does not correspond to the 210 kDa fragment of α _1_1.2 that arises via cleavage in the middle of the C-terminus, why then did Michailidis *et al*. not observe a doublet of 210 and 250 kDa in their hands with the ACC-003 / anti-L _II-III_ antibody? Perhaps differences in SDS-PAGE procedures might result in the 250 and 210 kDa size forms not being separated at all during their analysis and instead appear to migrate as one band at 250 kDa, analogous to our finding that the two size forms co-migrate as a single band in 11 and 13% gels. This is possible even in 8% gels as the electrophoresis period applied by Michailidis *et al*. was most likely shorter than in our hands. For the analysis of α _1_1.2 we reported here, the gel electrophoresis was extended to the point that the 60 kDa marker ran off the gel. Even with this protocol we see only partial separation of the 210 and 250 kDa forms of α _1_1.2 in our 9% gels ( Figure 4, Figure 5). With shorter running times, little to no separation is expected in 8% gels.

Immunocytochemical image analysis of ectopically expressed α _1_1.2 that carries a GFP tag at its cytosolic N-terminus and an HA tag in one of the extracellular loops of domain III for anti-HA antibody labeling of surface expressed Ca _V_1.2 was also used in the attempt to identify midchannel cleavage ( Michailidis *et al.*, 2014). The existence of clusters that only show GFP fluorescence is consistent with a substantial fraction of α _1_1.2 being intracellular where HA labeling is absent. The existence of often very large HA-immunoreactive red clusters lacking GFP signals was interpreted as evidence for separation of GFP and HA tags by proteolysis. If so, channel halves would completely dissociate and not remain close to each other as would be required for a channel to function with modified current conductance. Accordingly, the potential for separate N- and C-terminal portions of α _1_1.2 to form functional channels, as characterized by Michailidis *et al.* (2014), would either not be relevant in intact neurons if all of the cleaved channels dissociate or only apply to a small subpopulation of α _1_1.2; however the degree and function of spatial separation of N- and C-terminal α _1_1.2 fragments remains unclear.

Alternatively, rather than reflecting channel cleavage, the lack of detection of GFP signals in the HA-immunoreactive red clusters might be related to image acquisition or analysis. It is possible that the ectopically expressed α _1_1.2, together with the GFP tag signal, is much higher inside dendritic shafts than at their surfaces, resulting in a steep gradient toward the periphery. If so, when images are taken so that the GFP signal in the center of the shaft is in the dynamic range (i.e., fairly strong but not saturated), the peripheral signal would be much weaker. This appears to be the case in Figure 2B and 2D in the preceding work ( Michailidis *et al.*, 2014), where GFP seems to be mostly in the center of the dendrite and HA, as expected for surface labeling, at the periphery while sparing the center. In this manner, GFP could appear weak or absent in peripheral areas of dendrites where HA is mostly localized due to the surface labeling for HA. Such a scenario would provide one potential explanation for surface areas showing strong HA and weak GFP signals where actually a sizable fraction of uncleaved α _1_1.2 corresponding to the amount of HA signal might be present with the GFP signal at the surface appearing weak due to strong intracellular GFP signal.

Figure 2C in the Michailidis study ( Michailidis *et al.*, 2014) illustrates another potential scenario for dissociation of HA and GFP signals. This figure shows a long segment of the dendritic shaft that exhibits mostly HA and little if any GFP signal in. Even mild paraformaldehyde fixation can lead to permeabilization of 5–20 μm long segments of the dendritic plasma membrane and thereby expose sub-plasma membrane epitopes ( Taylor & Fallon, 2006; Watschinger *et al.*, 2008) (Matt and Hell, data not shown). Thus, it is conceivable that strong HA staining in this figure is paired with relatively strong suppression of GFP fluorescence in that segment as paraformaldehyde, which quenches GFP fluorescence, might have had preferential access to this region compared to the regions between the 0–2 and 12–14 μm marks where the GFP signal is much stronger. The strong HA staining in this dendritic segment could be surface labeling or intracellular HA staining of some sort of Cav1.2 clusters (perhaps reflecting a secretory compartment) due to antibody access induced by paraformaldehyde.

Evidence for the notion that HA staining likely yields much larger signals than GFP especially after fixation with paraformaldehyde is present in Figure 2D in this previous report ( Michailidis *et al.*, 2014). Here, protrusions are more strongly labeled by anti-HA staining than by GFP and shaft diameter appears much wider for HA than for GFP; these observations hint that the Ca _v_1.2-GFP signal in or near the plasma membrane is rather weak and largely from intracellular Ca _v_1.2. In this respect, it is surprising that there would be rather long segments of dendritic shaft that contain mostly HA and little if any GFP signal (as in Figure 2C of this publication).

We provide strong and clear evidence that the primary and major neuronal size forms of the α _1_1.2 subunit of Ca _V_1.2 are ~210 and 250 kDa in molecular mass. Based on detection of only a weak 150 kDa band by CNC1, ACC-003, and FP1 immunoblotting after immunoprecipitation with FP1 ( Figure 3 and Figure 6), it appears that a very small fraction of α _1_1.2 can be cleaved into 150 and 90 kDa fragments, which may remain to some degree associated with each other to form L-type channels of modified biophysical properties; however the prevalence of such proteolytic processing is certainly low (≤1%). It remains unclear what effects any limited mid-channel processing would have on overall L-type channel activity in neurons. It is possible that such processing of α _1_1.2 is more prominent in certain cell types or subcellular regions and could in fact lead to the change in channel properties described by Michailidis *et al*. Determining where and under what condition(s) changes might occur will further rouse interesting questions for the future.

## Data availability

The data referenced by this article are under copyright with the following copyright statement: Copyright: © 2018 Buonarati OR et al.

Data associated with the article are available under the terms of the Creative Commons Zero "No rights reserved" data waiver (CC0 1.0 Public domain dedication). 

**Dataset 1: Raw data supporting the findings presented in this study.** The raw data shows full size film images of probed membranes. Full size membranes resulting from transfer of full size gels were often vertically cut to separate replicate sets of samples typically separated by M _R_ markers for simultaneous probing of the different membrane fragments with different antibodies. For optimal resolution of the α _1_1.2 long and short forms, which exhibit high M _R_, gels were run until the 60 kDa M _R_ marker was either close to the very bottom of the gel or completely run off.

**Raw data for Figure 2. Determination of antibody specificity for α _1_1.2 with conditional α _1_1.2 KO mice.**

Original source images for Figure 2:

(A) Immunoblots of Triton X-100 extracts from conditional α _1_1.2 KO mice (KO) and litter matched WT mice using gels polymerized from 8% acrylamide. To ensure that there was no spill-over between lanes, in some gels one or more lanes were left empty as shown here for the middle lane labeled E in the right FP1 blot. To fully resolve α _1_1.2 short and long forms, the 100 kDa marker was run close to the bottom except in the right panel. In this experiment, electrophoresis of the same extracts used for α _1_1.2 immunoblotting was terminated before the dye front reached the bottom. Probing for β-actin showed that comparable amounts of protein were present in each extract from the different WT and KI mice.

(B) Ca _v_1.2 was immunoprecipitated from brain extracts from conditional KO and WT mice with the FP1 antibody before SDS-PAGE in gels polymerized from 6% acrylamide and immunoblotting with the indicated antibodies. To fully separate α _1_1.2 short and long forms, electrophoresis was performed until the 100 kDa marker was near the bottoms of the gels. For all antibodies, the ~210 and 250 kDa bands were nearly or completely absent in cKO samples.

**Raw data for Figure 3. Analysis of α _1_1.2 size forms by SDS-PAGE with increasing acrylamide concentrations.**

Original source images for Figure 3: Ca _v_1.2 was immunoprecipitated from mouse brain extracts (Triton X-100) with the FP1 antibody against α _1_1.2 before fractionation by SDS-PAGE in gels polymerized from 5, 7, 9, 11, and 13% acrylamide followed by immunoblotting with the indicated antibodies. Two different prestained marker protein sets were used to estimate M _R_.

**Raw data for Figure 4. Mouse and rat α _1_1.2 short forms co-migrate with α _1_1.2 truncated at residue 1800 in the middle of the c-terminus.**

Original source images for Figure 4: HEK293T cells were transfected with full length or truncated (Δ1800) α _1_1.2 plus α _2_δ _1_ and β _2a_. HEK293T cells and rat and mouse brain slices were extracted with 1% Triton X-100 before immunoprecipitation of α _1_1.2, SDS-PAGE in gels polymerized from 8% acrylamide, and immunoblotting with the indicated antibodies.

(A) The full length form of α _1_1.2 expressed in HEK293 cells migrated with an apparent M _R_ of 250 kDa and is detected by FP1, pS1700 and pS1928. Truncated Δ1800 α _1_1.2 migrated with an apparent M _R_ of 210 kDa and is detected by FP1 and pS1700 but not pS1928.

(B) The α _1_1.2 short and long form appear only partially resolved because the weak α _1_1.2 signals in HEK293 cell samples required long exposure times. The upper band as detected by CNC1 after FP1 immunoprecipitation from rat and mouse forebrain slices and cortical slices co-migrated with the full length form of α _1_1.2 expressed in HEK293 cells, while the lower band co-migrated with the truncated Δ1800 α _1_1.2 expressed in HEK293 cells. Sometimes, as seen here, a significant portion of the pore-forming subunit aggregated at the interface between stacking and resolving gels. This unresolved fraction (thick arrow) is not representative of its true molecular mass and not shown in the main figures.

**Raw data for Figure 5. Surface biotinylation labels α _1_1.2 size forms with apparent M _R_ > 200 kDa in rat cortical and forebrain slices.**

Original source images for Figure 5: Cortical and forebrain slices were surface biotinylated and solubilized before pulldown with NeutrAvidin Sepharose, SDS-PAGE in 8% acrylamide gels, and immunoblotting with CNC1 and FP1. Control reflects slices mock treated without Sulfo-NHS-SS-biotin to demonstrate specificity of pulldown. Twenty μL lysate was also directly loaded for comparison.

**Raw data for Figure 6. Differential recognition of the strong 150 kDa FP1 band in lysate and weak 150 kDa band by FP1, CNC1, and ACC-003 after IP of α _1_1.2 with FP1.**

Original source images for Figure 6: Immunoblots with CNC1 (A,B), FP1 (C), and ACC-003 (D,E) of Triton X-100 extracts from WT mice (lysate) and after immunoprecipitation with FP1 from cKO and WT mice. Gels were polymerized from 8% acrylamide. Note that a weak 150 kDa band is detected by CNC1, FP1, and ACC-003 after enrichment of α _1_1.2 by immunoprecipitation with FP1 but the strongly immunoreactive 150 kDa band detected by FP1 in lysate is not detectable by either CNC1 or ACC-003.

DOI, 10.5256/f1000research.11808.d168808 ( Buonarati *et al.*, 2017)

## References

[ref-1] Bavley CC Fischer DK Rizzo BK: Ca _v_1.2 channels mediate persistent chronic stress-induced behavioral deficits that are associated with prefrontal cortex activation of the p25/Cdk5-glucocorticoid receptor pathway. *Neurobiol Stress.* 2017;7:27–37. 10.1016/j.ynstr.2017.02.004 28289693 PMC5338724

[ref-2] Berkefeld H Sailer CA Bildl W: BK _Ca_-Cav channel complexes mediate rapid and localized Ca ^2+^-activated K ^+^ signaling. *Science.* 2006;314(5799):615–620. 10.1126/science.1132915 17068255

[ref-3] Bernard PB Castano AM Bayer KU: Necessary, but not sufficient: insights into the mechanisms of mGluR mediated long-term depression from a rat model of early life seizures. *Neuropharmacology.* 2014;84:1–12. 10.1016/j.neuropharm.2014.04.011 24780380 PMC4086946

[ref-4] Bolshakov VY Siegelbaum SA: Postsynaptic induction and presynaptic expression of hippocampal long-term depression. *Science.* 1994;264(5162):1148–52. 10.1126/science.7909958 7909958

[ref-5] Boric K Muñoz P Gallagher M: Potential adaptive function for altered long-term potentiation mechanisms in aging hippocampus. *J Neurosci.* 2008;28(32):8034–8039. 10.1523/JNEUROSCI.2036-08.2008 18685028 PMC2615232

[ref-6] Bunemann M Gerhardstein BL Gao T: Functional regulation of L-type calcium channels via protein kinase A-mediated phosphorylation of the beta(2) subunit. *J Biol Chem.* 1999;274(48):33851–33854. 10.1074/jbc.274.48.33851 10567342

[ref-7] Buonarati OR Henderson PB Murphy GG: Dataset 1 in: Proteolytic processing of the L-type Ca ^2+^ channel alpha _1_1.2 subunit in neurons. *F1000Research.* 2017 10.5256/f1000research.11808.d168808 PMC5531164 28781760

[ref-8] Cui Y Costa RM Murphy GG: Neurofibromin regulation of ERK signaling modulates GABA release and learning. *Cell.* 2008;135(3):549–560. 10.1016/j.cell.2008.09.060 18984165 PMC2673196

[ref-9] Davare MA Avdonin V Hall DD: A beta2 adrenergic receptor signaling complex assembled with the Ca ^2+^ channel Ca _v_1.2. *Science.* 2001;293(5527):98–101. 10.1126/science.293.5527.98 11441182

[ref-10] Davare MA Dong F Rubin CS: The A-kinase anchor protein MAP2B and cAMP-dependent protein kinase are associated with class C L-type calcium channels in neurons. *J Biol Chem.* 1999;274(42):30280–30287. 10.1074/jbc.274.42.30280 10514522

[ref-11] Davare MA Hell JW: Increased phosphorylation of the neuronal L-type Ca ^2+^ channel Ca _v_1.2 during aging. *Proc Natl Acad Sci U S A.* 2003;100(26):16018–16023. 10.1073/pnas.2236970100 14665691 PMC307685

[ref-12] Davare MA Horne MC Hell JW: Protein phosphatase 2A is associated with class C L-type calcium channels (Ca _v_1.2) and antagonizes channel phosphorylation by cAMP-dependent protein kinase. *J Biol Chem.* 2000;275(50):39710–39717. 10.1074/jbc.M005462200 10984483

[ref-13] De Jongh KS Murphy BJ Colvin AA: Specific phosphorylation of a site in the full-length form of the alpha 1 subunit of the cardiac L-type calcium channel by adenosine 3',5'-cyclic monophosphate-dependent protein kinase. *Biochemistry.* 1996;35(32):10392–10402. 10.1021/bi953023c 8756695

[ref-14] Dolmetsch RE Pajvani U Fife K: Signaling to the nucleus by an L-type calcium channel-calmodulin complex through the MAP kinase pathway. *Science.* 2001;294(5541):333–339. 10.1126/science.1063395 11598293

[ref-15] Dubel SJ Starr TV Hell J: Molecular cloning of the alpha-1 subunit of an omega-conotoxin-sensitive calcium channel. *Proc Natl Acad Sci U S A.* 1992;89(11):5058–5062. 10.1073/pnas.89.11.5058 1317580 PMC49228

[ref-16] Fuller MD Emrick MA Sadilek M: Molecular mechanism of calcium channel regulation in the fight-or-flight response. *Sci Signal.* 2010;3(141):ra70. 10.1126/scisignal.2001152 20876873 PMC3063709

[ref-17] Gao T Bunemann M Gerhardstein BL: Role of the C terminus of the alpha 1C (CaV1.2) subunit in membrane targeting of cardiac L-type calcium channels. *J Biol Chem.* 2000;275(33):25436–25444. 10.1074/jbc.M003465200 10816591

[ref-18] Gao T Cuadra AE Ma H: C-terminal fragments of the alpha 1C (Ca _V_1.2) subunit associate with and regulate L-type calcium channels containing C-terminal-truncated alpha 1C subunits. *J Biol Chem.* 2001;276(24):21089–21097. 10.1074/jbc.M008000200 11274161

[ref-19] Graef IA Mermelstein PG Stankunas K: L-type calcium channels and GSK-3 regulate the activity of NF-ATc4 in hippocampal neurons. *Nature.* 1999;401(6754):703–708. 10.1038/44378 10537109

[ref-20] Grover LM Teyler TJ: Two components of long-term potentiation induced by different patterns of afferent activation. *Nature.* 1990;347(6292):477–479. 10.1038/347477a0 1977084

[ref-21] Hall DD Dai S Tseng PY: Competition between α-actinin and Ca ^2+^-calmodulin controls surface retention of the L-type Ca ^2+^ channel Ca _V_1.2. *Neuron.* 2013;78(3):483–497. 10.1016/j.neuron.2013.02.032 23664615 PMC4570828

[ref-22] Hall DD Davare MA Shi M: Critical role of cAMP-dependent protein kinase anchoring to the L-type calcium channel Ca _v_1.2 via A-kinase anchor protein 150 in neurons. *Biochemistry.* 2007;46(6):1635–1646. 10.1021/bi062217x 17279627

[ref-23] Hall DD Feekes JA Arachchige Don AS: Binding of protein phosphatase 2A to the L-type calcium channel Ca _v_1.2 next to Ser1928, its main PKA site, is critical for Ser1928 dephosphorylation. *Biochemistry.* 2006;45(10):3448–3459. 10.1021/bi051593z 16519540

[ref-24] Hell JW Westenbroek RE Breeze LJ: N-methyl-D-aspartate receptor-induced proteolytic conversion of postsynaptic class C L-type calcium channels in hippocampal neurons. *Proc Natl Acad Sci U S A.* 1996;93(8):3362–3367. 10.1073/pnas.93.8.3362 8622942 PMC39613

[ref-25] Hell JW Westenbroek RE Warner C: Identification and differential subcellular localization of the neuronal class C and class D L-type calcium channel alpha 1 subunits. *J Cell Biol.* 1993a;123(4):949–962. 10.1083/jcb.123.4.949 8227151 PMC2200142

[ref-26] Hell JW Yokoyama CT Breeze LJ: Phosphorylation of presynaptic and postsynaptic calcium channels by cAMP-dependent protein kinase in hippocampal neurons. *EMBO J.* 1995;14(13):3036–3044. 7621818 10.1002/j.1460-2075.1995.tb07306.x PMC394364

[ref-27] Hell JW Yokoyama CT Wong ST: Differential phosphorylation of two size forms of the neuronal class C L-type calcium channel alpha 1 subunit. *J Biol Chem.* 1993b;268(26):19451–19457. 8396138

[ref-28] Hulme JT Westenbroek RE Scheuer T: Phosphorylation of serine 1928 in the distal C-terminal domain of cardiac Ca _V_1.2 channels during beta1-adrenergic regulation. *Proc Natl Acad Sci U S A.* 2006a;103(44):16574–16579. 10.1073/pnas.0607294103 17053072 PMC1637623

[ref-29] Hulme JT Yarov-Yarovoy V Lin TW: Autoinhibitory control of the Ca _V_1.2 channel by its proteolytically processed distal C-terminal domain. *J Physiol.* 2006b;576(Pt 1):87–102. 10.1113/jphysiol.2006.111799 16809371 PMC1995633

[ref-30] Kochlamazashvili G Henneberger C Bukalo O: The extracellular matrix molecule hyaluronic acid regulates hippocampal synaptic plasticity by modulating postsynaptic L-type Ca ^2+^ channels. *Neuron.* 2010;67(1):116–128. 10.1016/j.neuron.2010.05.030 20624596 PMC3378029

[ref-31] Li H Pink MD Murphy JG: Balanced interactions of calcineurin with AKAP79 regulate Ca ^2+^-calcineurin-NFAT signaling. *Nat Struct Mol Biol.* 2012;19(3):337–345. 10.1038/nsmb.2238 22343722 PMC3294036

[ref-32] Liao P Yu D Hu Z: Alternative splicing generates a novel truncated Ca _v_1.2 channel in neonatal rat heart. *J Biol Chem.* 2015;290(14):9262–9272. 10.1074/jbc.M114.594911 25694430 PMC4423710

[ref-33] Ma H Groth RD Cohen SM: γCaMKII shuttles Ca ^2+^/CaM to the nucleus to trigger CREB phosphorylation and gene expression. *Cell.* 2014;159(2):281–294. 10.1016/j.cell.2014.09.019 25303525 PMC4201038

[ref-34] Marrion NV Tavalin SJ: Selective activation of Ca ^2+^-activated K ^+^ channels by co-localized Ca ^2+^ channels in hippocampal neurons. *Nature.* 1998;395(6705):900–905. 10.1038/27674 9804423

[ref-35] Marshall MR Clark JP 3rd Westenbroek R: Functional roles of a C-terminal signaling complex of Ca _V_1 channels and A-kinase anchoring protein 15 in brain neurons. *J Biol Chem.* 2011;286(14):12627–12639. 10.1074/jbc.M110.175257 21224388 PMC3069463

[ref-36] Michailidis IE Abele-Henckels K Zhang WK: Age-related homeostatic midchannel proteolysis of neuronal L-type voltage-gated Ca ^2+^ channels. *Neuron.* 2014;82(5):1045–1057. 10.1016/j.neuron.2014.04.017 24908485 PMC4052215

[ref-37] Mikami A Imoto K Tanabe T: Primary structure and functional expression of the cardiac dihydropyridine-sensitive calcium channel. *Nature.* 1989;340(6230):230–233. 10.1038/340230a0 2474130

[ref-38] Moosmang S Haider N Klugbauer N: Role of hippocampal Ca _v_1.2 Ca ^2+^ channels in NMDA receptor-independent synaptic plasticity and spatial memory. *J Neurosci.* 2005;25(43):9883–9892. 10.1523/JNEUROSCI.1531-05.2005 16251435 PMC6725564

[ref-39] Murphy JG Sanderson JL Gorski JA: AKAP-anchored PKA maintains neuronal L-type calcium channel activity and NFAT transcriptional signaling. *Cell Rep.* 2014;7(5):1577–1588. 10.1016/j.celrep.2014.04.027 24835999 PMC4136445

[ref-40] Patriarchi T Qian H Di Biase V: Phosphorylation of Ca _v_1.2 on S1928 Uncouples the L-type Ca ^2+^ Channel from the β2 Adrenergic Receptor. *EMBO J.* 2016;35(12):1330–1345. 10.15252/embj.201593409 27103070 PMC4910527

[ref-41] Qian H Patriarchi T Price JL: Phosphorylation of Ser ^1928^ mediates the enhanced activity of the L-type Ca ^2+^ channel Cav1.2 by the β _2_-adrenergic receptor in neurons. *Sci Signal.* 2017;10(463): Pii: eaaf9659. 10.1126/scisignal.aaf9659 28119465 PMC5310946

[ref-42] Seisenberger C Specht V Welling A: Functional embryonic cardiomyocytes after disruption of the L-type alpha _1C_ ( *Ca _v_1.2*) calcium channel gene in the mouse. *J Biol Chem.* 2000;275(50):39193–39199. 10.1074/jbc.M006467200 10973973

[ref-43] Sinnegger-Brauns MJ Hetzenauer A Huber IG: Isoform-specific regulation of mood behavior and pancreatic beta cell and cardiovascular function by L-type Ca ^2+^ channels. *J Clin Invest.* 2004;113(10):1430–1439. 10.1172/JCI20208 15146240 PMC406526

[ref-44] Snutch TP Tomlinson WJ Leonard JP: Distinct calcium channels are generated by alternative splicing and are differentially expressed in the mammalian CNS. *Neuron.* 1991;7(1):45–57. 10.1016/0896-6273(91)90073-9 1648941

[ref-45] Splawski I Timothy KW Sharpe LM: Ca _V_1.2 calcium channel dysfunction causes a multisystem disorder including arrhythmia and autism. *Cell.* 2004;119(1):19–31. 10.1016/j.cell.2004.09.011 15454078

[ref-46] Taylor AB Fallon JR: Dendrites contain a spacing pattern. *J Neurosci.* 2006;26(4):1154–1163. 10.1523/JNEUROSCI.4424-05.2006 16436602 PMC6674572

[ref-47] Tippens AL Pare JF Langwieser N: Ultrastructural evidence for pre- and postsynaptic localization of Ca _v_1.2 L-type Ca ^2+^ channels in the rat hippocampus. *J Comp Neurol.* 2008;506(4):569–583. 10.1002/cne.21567 18067152

[ref-48] Tseng PY Henderson PB Hergarden AC: α-Actinin Promotes Surface Localization and Current Density of the Ca ^2+^ Channel Ca _V_1.2 by Binding to the IQ Region of the α1 Subunit. *Biochemistry.* 2017;56(28):3669–3681. 10.1021/acs.biochem.7b00359 28613835 PMC5704914

[ref-49] Watschinger K Horak SB Schulze K: Functional properties and modulation of extracellular epitope-tagged Ca _V_2.1 voltage-gated calcium channels. *Channels (Austin).* 2008;2(6):461–473. 10.4161/chan.2.6.6793 18797193 PMC3942855

[ref-50] Wei X Neely A Lacerda AE: Modification of Ca ^2+^ channel activity by deletions at the carboxyl terminus of the cardiac alpha 1 subunit. *J Biol Chem.* 1994;269(3):1635–1640. 7507480

[ref-51] Westenbroek RE Hell JW Warner C: Biochemical properties and subcellular distribution of an N-type calcium channel alpha 1 subunit. *Neuron.* 1992;9(6):1099–1115. 10.1016/0896-6273(92)90069-P 1334419

[ref-52] Wheeler DG Groth RD Ma H: Ca _V_1 and Ca _V_2 channels engage distinct modes of Ca ^2+^ signaling to control CREB-dependent gene expression. *Cell.* 2012;149(5):1112–1124. 10.1016/j.cell.2012.03.041 22632974 PMC3654514

[ref-53] White JA McKinney BC John MC: Conditional forebrain deletion of the L-type calcium channel Ca _V_1.2 disrupts remote spatial memories in mice. *Learn Mem.* 2008;15(1):1–5. 10.1101/lm.773208 18174367

[ref-54] Zhu Y Romero MI Ghosh P: Ablation of NF1 function in neurons induces abnormal development of cerebral cortex and reactive gliosis in the brain. *Genes Dev.* 2001;15(7):859–876. 10.1101/gad.862101 11297510 PMC312666

