# Supplementary material for: Proteolytic processing of the L-type Ca 2+ channel alpha 11.2 subunit in neurons
Source: F1000Res. 2018 Aug 17;6:1166. Originally published 2017 Jul 21. [Version 2] doi: 10.12688/f1000research.11808.2 (PMC5531164; doi:10.12688/f1000research.11808.2)

Raw data for Figure 1

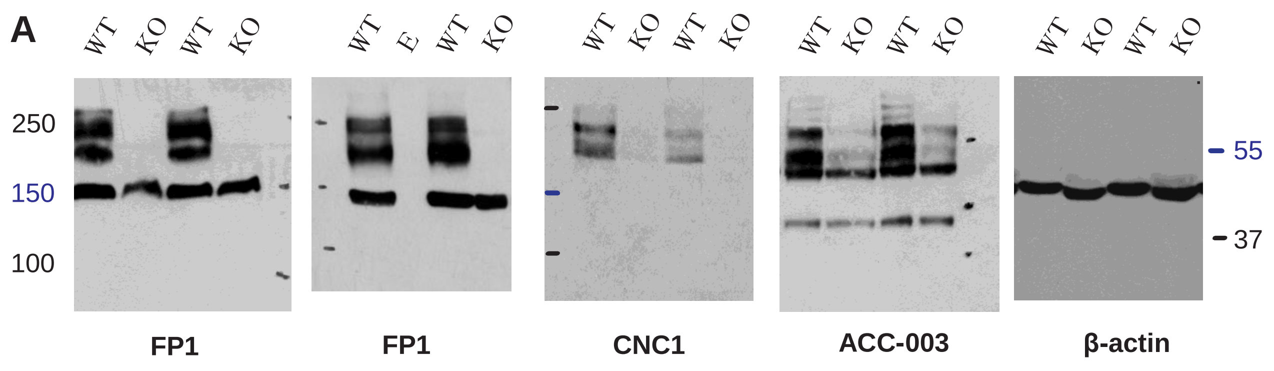

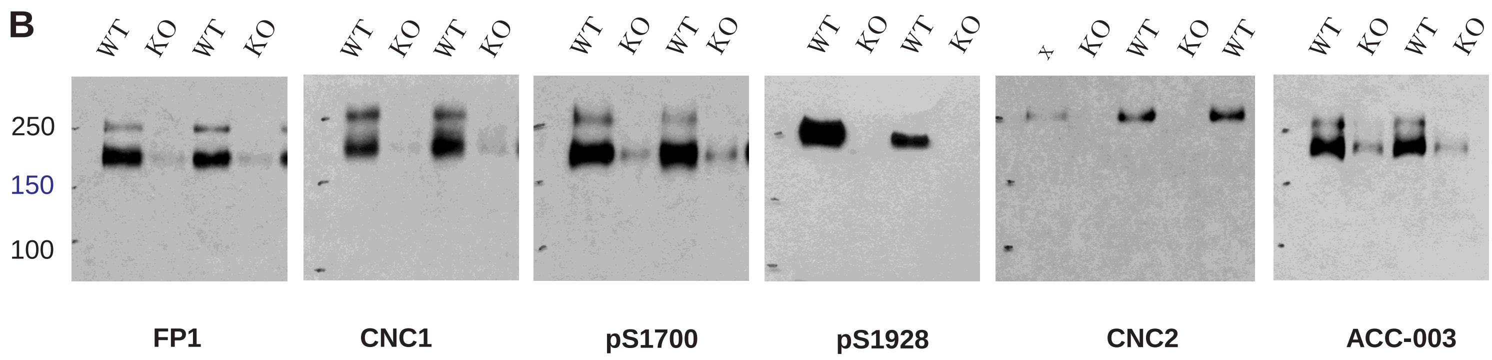

Raw data for Figure 2

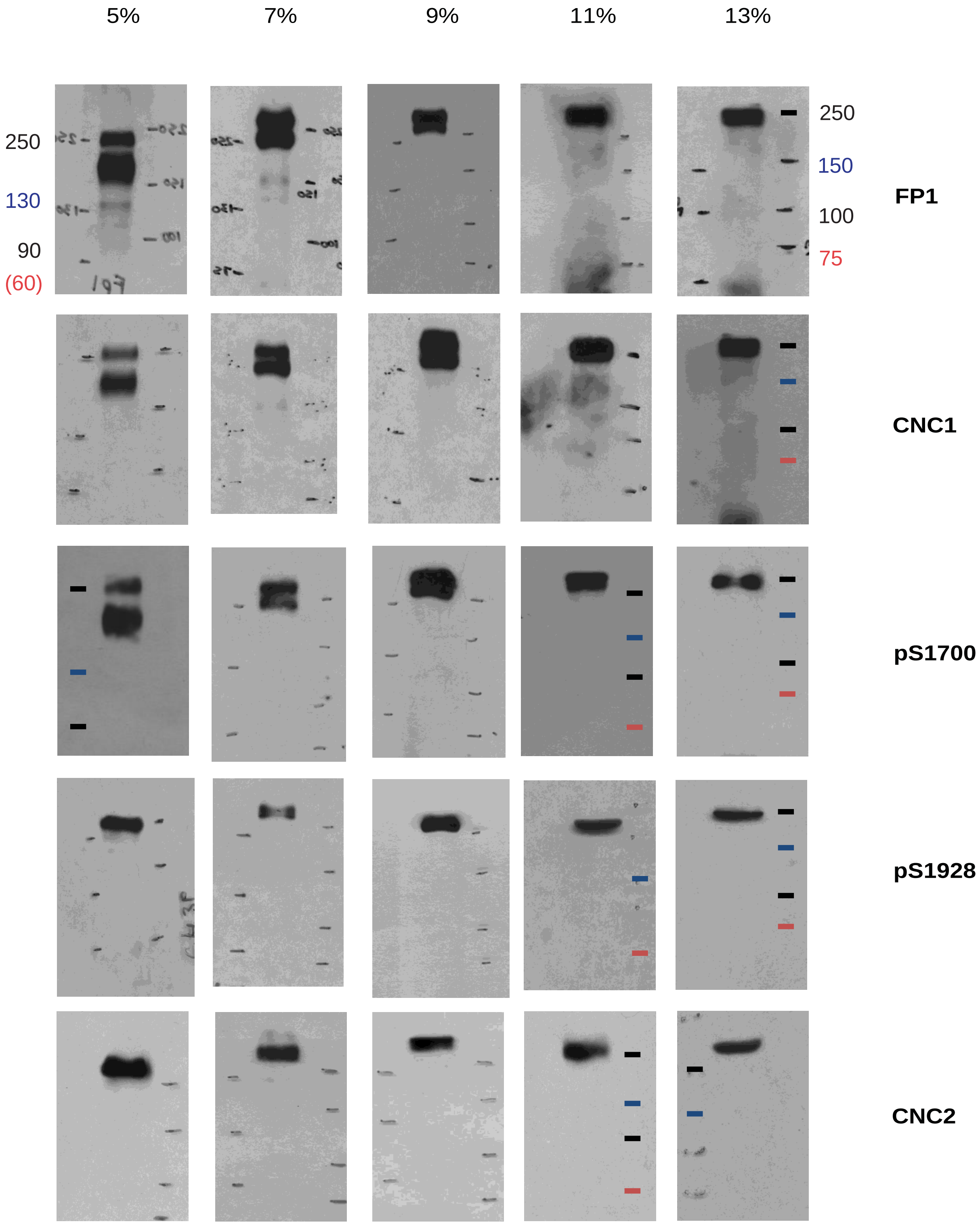

Raw data for Figure 3

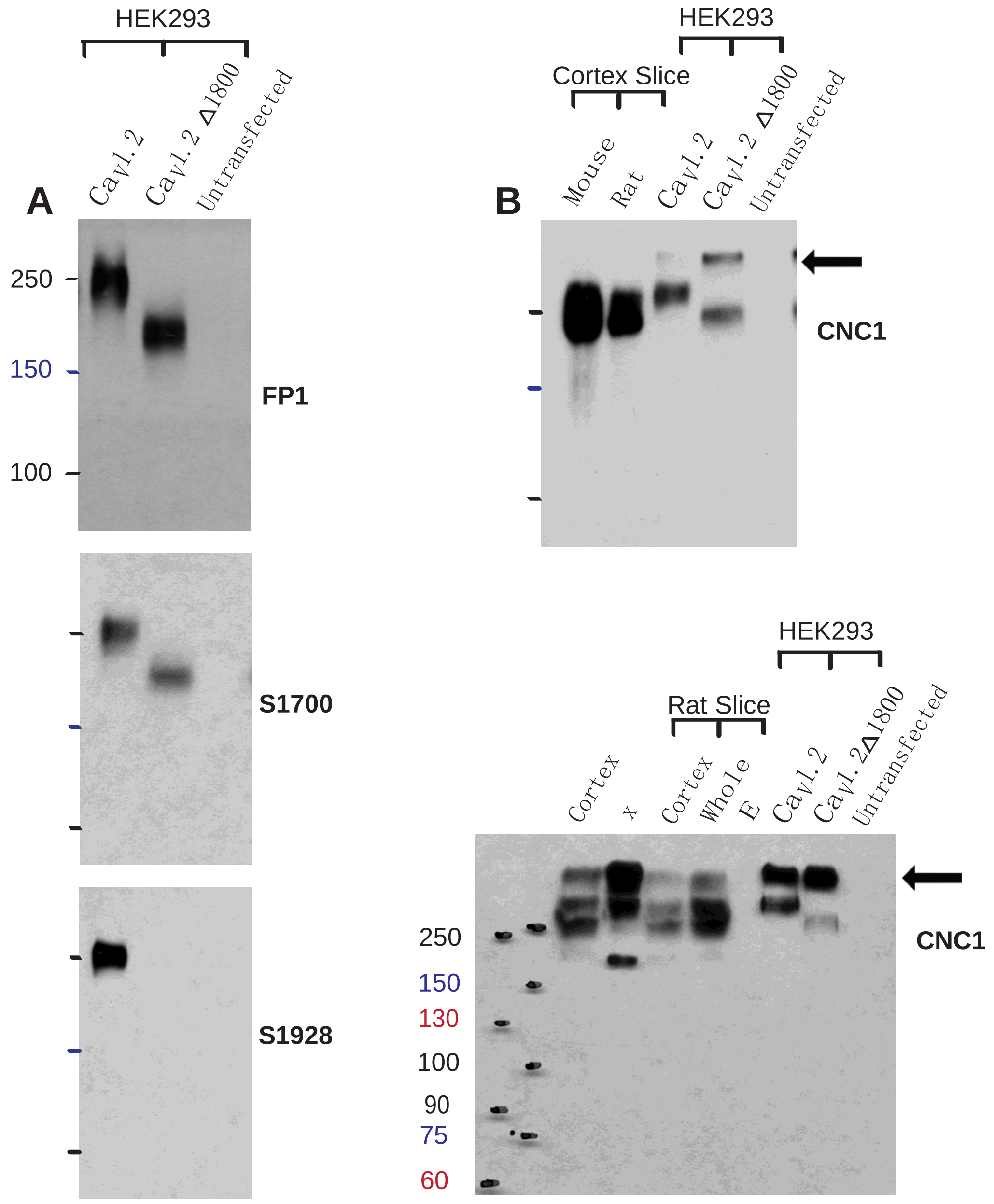

Raw data for Figure 4

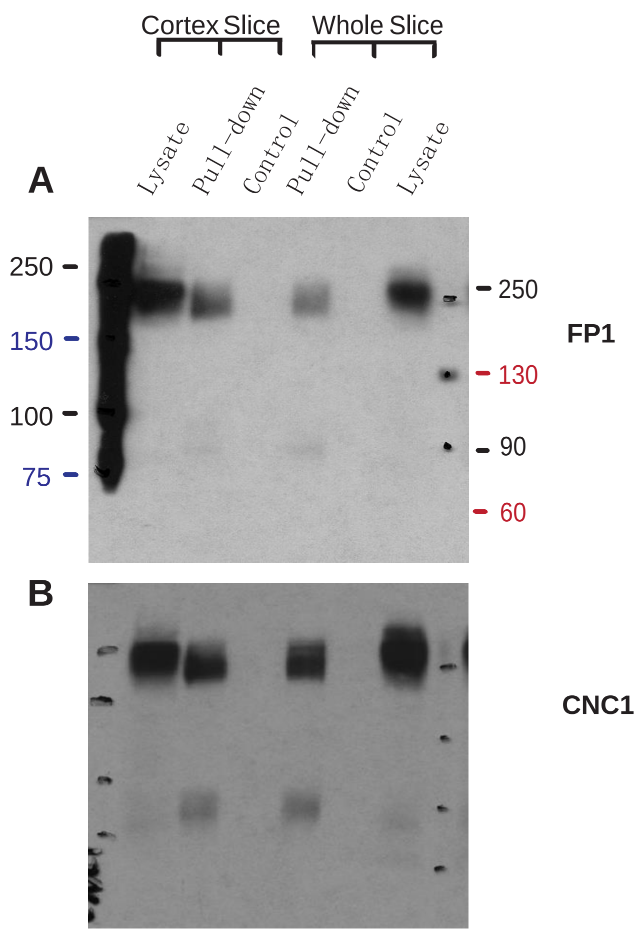

Raw data for Figure 5

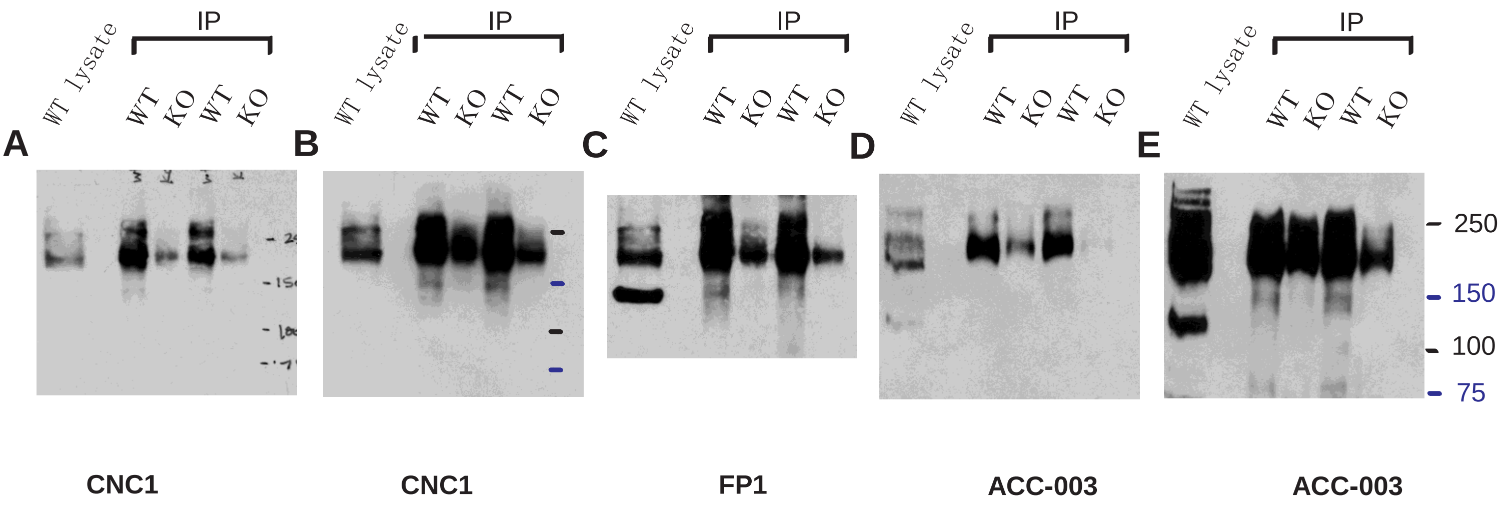

Supplement: Raw data supporting the findings presented in this study — The raw data shows full size film images of probed membranes. Full size membranes resulting from transfer of full size gels were often vertically cut to separate replicate sets of samples typically separated by M R markers for simultaneous probing of the different membrane fragments with different antibodies. For optimal resolution of the α 11.2 long and short forms, which exhibit high M R, gels were run until the 60 kDa M R marker was either close to the very bottom of the gel or completely run off. Raw data for Figure 2. Determination of antibody specificity for α 11.2 with conditional α 11.2 KO mice. Original source images for Figure 2: (A) Immunoblots of Triton X-100 extracts from conditional α 11.2 KO mice (KO) and litter matched WT mice using gels polymerized from 8% acrylamide. To ensure that there was no spill-over between lanes, in some gels one or more lanes were left empty as shown here for the middle lane labeled E in the right FP1 blot. To fully resolve α 11.2 short and long forms, the 100 kDa marker was run close to the bottom except in the right panel. In this experiment, electrophoresis of the same extracts used for α 11.2 immunoblotting was terminated before the dye front reached the bottom. Probing for b-actin showed that comparable amounts of protein were present in each extract from the different WT and KI mice. (B) Ca v1.2 was immunoprecipitated from brain extracts from conditional KO and WT mice with the FP1 antibody before SDS-PAGE in gels polymerized from 6% acrylamide and immunoblotting with the indicated antibodies. To fully separate α 11.2 short and long forms, electrophoresis was performed until the 100 kDa marker was near the bottoms of the gels. For all antibodies, the ~210 and 250 kDa bands were nearly or completely absent in cKO samples. Raw data for Figure 3. Analysis of α 11.2 size forms by SDS-PAGE with increasing acrylamide concentrations. Original source images for Figure 3: Ca v1.2 was immunoprecipitated from mouse brain extract [file f1000research-6-17419-s0000.tgz › 221db438-aa9b-4a84-9c6a-050aafe7a2ef_Raw_data_.pdf]
