# Supplementary material for: Proteolytic processing of the L-type Ca 2+ channel alpha 11.2 subunit in neurons
Source: F1000Res. 2018 Aug 17;6:1166. Originally published 2017 Jul 21. [Version 2] doi: 10.12688/f1000research.11808.2 (PMC5531164; doi:10.12688/f1000research.11808.2)
Supplement: Peer review history

# Peer review: Referee response for version 2

**DOI:** 10.5256/f1000research.17419.r37331

**Published:** 2018-08-20

**Authors:** Mark L. Dell’Acqua

**Affiliations:** 1 Department of Pharmacology, University of Colorado Denver, Denver, CO, USA

No new comments.

I have read this submission. I believe that I have an appropriate level of expertise to confirm that it is of an acceptable scientific standard.

# Peer review: Referee response for version 2

**DOI:** 10.5256/f1000research.17419.r37330

**Published:** 2018-08-20

**Authors:** Annette Dolphin

**Affiliations:** 1 Department of Neuroscience, Physiology and Pharmacology, University College London, London, UK

The additional Figures extend these Results, and  reinforce the authors' conclusions.  It is an object lesson in careful and insightful work.  Congratulations to the authors.

I have read this submission. I believe that I have an appropriate level of expertise to confirm that it is of an acceptable scientific standard.

# Peer review: Referee response for version 1

**DOI:** 10.5256/f1000research.12761.r24429

**Published:** 2017-08-16

**Authors:** Mark L. Dell’Acqua

**Affiliations:** 1 Department of Pharmacology, University of Colorado Denver, Denver, CO, USA

Buonarati *et al. *have performed a very rigorous biochemical analysis using 6 different antibodies and conditional knockout (KO) mice controls to convincingly demonstrate that the major forms of the L-channel Ca _V_1.2 pore forming subunit present in mouse and rat brain are full-length channels of ~250 kDa and C-terminally truncated channels of ~210 kDa. Importantly, the authors' exhaustive analysis using  immunoblotting of whole cell extracts as well as immunoprecipitation or surface biotinylation followed by immunoblotting provides no evidence for Ca _V_1.2 mid-channel cleavage that would produce ~150 kDa and ~90 kDa bands as reported in Michailidis *et al.*, 2014 ^1^.  In particular, by employing neuron-specific Ca _V_1.2 conditional KO mice as controls in conjunction with multiple antibodies spanning the N- to C-terminal regions of Ca _V_1.2, Buonarati *et al.* demonstrate that the previously reported ~150 kDa and ~90 kDa bands are most likely protein products unrelated to Ca _V_1.2 that cross-react with some but not all Ca _V_1.2  antibodies.

 Overall, this study is extremely well executed and thoughtfully discussed to provide a very valuable addition to the L-channel literature.  I only have one minor question/comment for the authors.

1. The authors cite previous publications from the Hosey and Catterall groups showing that the ~40 kDa distal C-terminal fragment produced by cleavage of Ca _V_1.2 remains associated with the ~210 kDa fragment to regulate channel function (Fuller *et al.*, 2010 ^2^; Gao *et al.*, 2001 ^3^; Hulme *et al.*, 2006b ^4^). However, these prior studies were focused on Ca _V_1.2 cleavage in muscle cells and primarily relied on reconstitution of the association between the 40 kDa distal C-terminal fragment and the  ~210 kDa body of the channel by heterologous expression.  Thus, it would be interesting if the authors could determine if the ~40 kDA distal C-terminal fragment of Ca _V_1.2 is also present in mouse and rat brain and whether this fragment can co-immunoprecipitate with the ~210 kDa fragment. Such additional information could be valuable in understanding the differences in PKA regulation of Ca _V_1.2 channels through phosphorylation at S1928 in the distal C-terminus versus S1700 in the proximal C-terminus recently reported by the authors for neurons ( Qian *et al.*, 2017 ^5^) compared to earlier work by others for cardiac myocytes (Fuller *et al.*, 2010 ^2^; Moosmang *et al.*, 2005 ^6^).   

I have read this submission. I believe that I have an appropriate level of expertise to confirm that it is of an acceptable scientific standard.

## References

[1] MichailidisIEAbele-HenckelsKZhangWKLinBYuYGeymanLSEhlersMDPnevmatikakisEAYangJ: Age-related homeostatic midchannel proteolysis of neuronal L-type voltage-gated Ca²⁺ channels. *Neuron*.2014;82(5) : 10.1016/j.neuron.2014.04.017 1045-57 10.1016/j.neuron.2014.04.017 24908485PMC4052215

[2] FullerMDEmrickMASadilekMScheuerTCatterallWA: Molecular mechanism of calcium channel regulation in the fight-or-flight response. *Sci Signal*.2010;3(141) : 10.1126/scisignal.2001152 ra70 10.1126/scisignal.2001152 20876873PMC3063709

[3] GaoTCuadraAEMaHBunemannMGerhardsteinBLChengTEickRTHoseyMM: C-terminal fragments of the alpha 1C (CaV1.2) subunit associate with and regulate L-type calcium channels containing C-terminal-truncated alpha 1C subunits. *J Biol Chem*.2001;276(24) : 10.1074/jbc.M008000200 21089-97 10.1074/jbc.M008000200 11274161

[4] HulmeJTYarov-YarovoyVLinTWScheuerTCatterallWA: Autoinhibitory control of the CaV1.2 channel by its proteolytically processed distal C-terminal domain. *J Physiol*.2006;576(Pt 1) : 10.1113/jphysiol.2006.111799 87-102 10.1113/jphysiol.2006.111799 16809371PMC1995633

[5] QianHPatriarchiTPriceJLMattLLeeBNieves-CintrónMBuonaratiORChowdhuryDNanouENystoriakMACatterallWAPoomvanichaMHofmannFNavedoMFHellJW: Phosphorylation of Ser1928 mediates the enhanced activity of the L-type Ca2+ channel Cav1.2 by the β2-adrenergic receptor in neurons. *Sci Signal*.2017;10(463) : 10.1126/scisignal.aaf9659 10.1126/scisignal.aaf9659 28119465PMC5310946

[6] MoosmangSHaiderNKlugbauerNAdelsbergerHLangwieserNMüllerJStiessMMaraisESchullaVLacinovaLGoebbelsSNaveKAStormDRHofmannFKleppischT: Role of hippocampal Cav1.2 Ca2+ channels in NMDA receptor-independent synaptic plasticity and spatial memory. *J Neurosci*.2005;25(43) : 10.1523/JNEUROSCI.1531-05.2005 9883-92 10.1523/JNEUROSCI.1531-05.2005 16251435PMC6725564

# Peer review: Referee response for version 1

**DOI:** 10.5256/f1000research.12761.r24425

**Published:** 2017-07-26

**Authors:** Jörg Striessnig

**Affiliations:** 1 Department of Pharmacology and Toxicology, University of Innsbruck, Innsbruck, Austria

Buonarati *et al.* provide a long overdue answer to a previous publication in NEURON (their ref Michailidis *et al,*. 2014) which reported the possibility of proteolytic processing of Cav1.2 Ca ^2+^ channel a1-subunits within the II-III cytoplasmic loop giving rise to a 150 kDa polypeptide. Based on this finding further experiments suggested a functional role of this "midchannel proteolysis", in particular a homeostatic feedback regulation affecting neuronal signaling. However, these findings were heavily criticized by experts familiar with the biochemistry of Cav1.2 channels because essential controls for the existence of such a 150 kDa polypeptide were missing in the publication of Michailidis *et al.* (2014) ^1^.

 First, the widely established and accepted golden standard to verify the specificity of antibodies is to use identically prepared samples from knockout animals. In the case of Cav1.2 these animals are widely available from several groups and frozen brains can be easily shipped on ice. Second, the molecular mass standards used in the paper to provide convincing support for such a far-reaching conclusion are insufficiently described. Third, no efforts have been made to systematically verify the mass of the 150 kDa polypeptide by a recombinant HEK-293 cell – expressed protein of the proposed sequence (also standard in the field). Given the potential impact of the existence of midchannel proteolysis for the Ca ^2+^ channel field it is difficult to rationalize why reviewers of this paper have not insisted on these simple controls.

 Fortunately, Buonarati and colleagues have now performed exactly these experiments at the highest possible technical level. And it is therefore not surprising that they cannot confirm a significant level of midchannel proteolysis in mouse or rat brain, even under conditions very similar to those used by Michailidis *et al*.. The experiments were repeated with 6 different antibodies, carefully selected molecular mass markers  and also using different polyacrylamide concentrations to account for the possible aberrant migration of these large membrane proteins. Their findings are clearly presented and nicely discussed and offer several potential explanations for the discrepant findings.

 In summary, this publication is an excellent contribution to this field and will hopefully end the discussion about midchannel proteolysis.

I have read this submission. I believe that I have an appropriate level of expertise to confirm that it is of an acceptable scientific standard.

## References

[1] MichailidisIEAbele-HenckelsKZhangWKLinBYuYGeymanLSEhlersMDPnevmatikakisEAYangJ: Age-related homeostatic midchannel proteolysis of neuronal L-type voltage-gated Ca²⁺ channels. *Neuron*.2014;82(5) : 10.1016/j.neuron.2014.04.017 1045-57 10.1016/j.neuron.2014.04.017 24908485PMC4052215

# Peer review: Referee response for version 1

**DOI:** 10.5256/f1000research.12761.r24428

**Published:** 2017-07-26

**Authors:** Annette Dolphin

**Affiliations:** 1 Department of Neuroscience, Physiology and Pharmacology, University College London, London, UK

This paper describes an attempt at replication of the results of Michailidis *et al*, regarding the existence and relevance of mid-channel proteolysis of Ca _V_1.2, previously described as a homeostatic mechanism to regulate channel activity (Michailidis *et al*., 2014 ^1^).  Mid channel proteolysis was shown in that paper to occur in the II-III linker and to result in prominent 150 kDa and 90 kDa bands.

 The careful work of Buonarati *et al *describes here the use of multiple different Ca _V_1.2 antibodies and % gels, and shows conclusively that the most prominent size forms are 250 and 210 kDa, relating to full length and C-terminally cleaved Ca _V_1.2 channels in brain tissue.  A 150 kDa band was also observed with two of the antibodies, but was still as prominent in KO mouse tissue, indicating it is not a Ca _V_1.2-related fragment. The other antibodies identified only very minor 150 kDa bands, estimated to be <1% of the total, and the authors conclude mid-channel proteolysis is minimal in brain tissue.

 In the Discussion the authors describe several possibilities that could account for the disparity of results, including % gels used and gel run times, as well as antibody specificity, leading to mis-identification of bands. They also critique another result in the original paper, partial lack of co-localization of an N-terminal-GFP tag and an extracellular HA tag in Ca _V_1.2, which was also originally attributed to mid-channel proteolysis.  They point out that GFP is quenched by paraformaldehyde and fixation also induces partial permeabilization of hippocampal neurons in culture. In my view this is a completely reasonable comment, and probably should have been picked up by the original referees.

 In doing this painstaking study, the authors have also done a great service to the community by comparing multiple different antibodies to Ca _V_1.2.

**Minor comments**.

1. Do the authors have any clues about the 150 kDa band, is it a contaminant from blood?  Have the authors ever perfused mice or rats with ice-cold saline before harvesting the brain, to determine whether it is reduced?
2. Fig. 5B, identify the MW markers on the left.
3. Fig 6 might be easier to grasp rapidly if the authors added IB: CNC1 on the left, and put IP next to all the Ab labels on the bottom of each blot.
4. I may have missed how the authors quantified any mid-channel proteolytic processing to be ~ 1% (page 12).
5. In the Abstract, I suggest two changes:
  - line 5 change “Recent work suggests..” to “However, recent work further suggests”
  - last line change “at best” to “at most”

I have read this submission. I believe that I have an appropriate level of expertise to confirm that it is of an acceptable scientific standard.

## References

[1] MichailidisIEAbele-HenckelsKZhangWKLinBYuYGeymanLSEhlersMDPnevmatikakisEAYangJ: Age-related homeostatic midchannel proteolysis of neuronal L-type voltage-gated Ca²⁺ channels. *Neuron*.2014;82(5) : 10.1016/j.neuron.2014.04.017 1045-57 10.1016/j.neuron.2014.04.017 24908485PMC4052215
